# Viral community-wide auxiliary metabolic genes differ by lifestyles, habitats, and hosts

**DOI:** 10.1186/s40168-022-01384-y

**Published:** 2022-11-05

**Authors:** Xiao-Qing Luo, Pandeng Wang, Jia-Ling Li, Manzoor Ahmad, Li Duan, Ling-Zi Yin, Qi-Qi Deng, Bao-Zhu Fang, Shan-Hui Li, Wen-Jun Li

**Affiliations:** 1 State Key Laboratory of Biocontrol, Guangdong Provincial Key Laboratory of Plant Resources and Southern Marine Science and Engineering Guangdong Laboratory (Zhuhai), School of Life Sciences, Sun Yat-Sen University, Guangzhou, 510275 People’s Republic of China; 2 School of Ecology, Shenzhen Campus of Sun Yat-Sen University, Shenzhen, 518107 People’s Republic of China; 3 State Key Laboratory of Desert and Oasis Ecology, Xinjiang Institute of Ecology and Geography, Chinese Academy of Sciences, Urumqi, 830011 People’s Republic of China

**Keywords:** Auxiliary metabolic genes (AMGs), Viral lifestyles, Metagenomes and metatranscriptomes, Biogeochemical cycles

## Abstract

**Background:**

Viral-encoded auxiliary metabolic genes (AMGs) are important toolkits for modulating their hosts’ metabolisms and the microbial-driven biogeochemical cycles. Although the functions of AMGs have been extensively reported in numerous environments, we still know little about the drivers that shape the viral community-wide AMG compositions in natural ecosystems. Exploring the drivers of viral community-wide AMG compositions is critical for a deeper understanding of the complex interplays among viruses, hosts, and the environments.

**Results:**

Here, we investigated the impact of viral lifestyles (i.e., lytic and lysogenic), habitats (i.e., water, particle, and sediment), and prokaryotic hosts on viral AMG profiles by utilizing metagenomic and metatranscriptomic techniques. We found that viral lifestyles were the most important drivers, followed by habitats and host identities. Specifically, irrespective of what habitats viruses came from, lytic viruses exhibited greater AMG diversity and tended to encode AMGs for chaperone biosynthesis, signaling proteins, and lipid metabolism, which could boost progeny reproduction, whereas temperate viruses were apt to encode AMGs for host survivability. Moreover, the lytic and temperate viral communities tended to mediate the microbial-driven biogeochemical cycles, especially nitrogen metabolism, in different manners via AMGs. When focusing on each lifestyle, we further found clear dissimilarity in AMG compositions between water and sediment, as well the divergent AMGs encoded by viruses infecting different host orders.

**Conclusions:**

Overall, our study provides a first systematic characterization of the drivers of viral community-wide AMG compositions and further expands our knowledge of the distinct interactions of lytic and temperate viruses with their prokaryotic hosts from an AMG perspective, which is critical for understanding virus-host-environment interactions in natural conditions.

Video Abstract

**Supplementary Information:**

The online version contains supplementary material available at 10.1186/s40168-022-01384-y.

## Introduction

Viruses have been found in almost all explored ecosystems and are the most abundant and diverse biological entities on Earth [1]. Viruses that infect bacteria (phages) or archaea (archaeal viruses) can strongly affect microbial metabolisms, diversity, and evolution via auxiliary metabolic genes (AMGs) [1–3]. AMGs are highly prevalent in viral genomes and have been reported to be involved in diverse functions, including nutrient metabolism, transportation, bacterial motility, and biofilm formation [4, 5]. Based on their functions, AMGs could be grouped into two classes [5]. Class I AMGs encompass genes that are directly involved in the metabolic pathways defined by Kyoto Encyclopedia of Genes and Genomes (KEGG), while genes that perform peripheral roles in metabolisms belong to class II AMGs [5]. With the improvement of metagenomic and viromic analyses [6–8], numerous novel AMGs were successively uncovered. For instance, active AMGs that are associated with glycoside hydrolysis (class I) [6] and transmembrane substrates transportation (class II) [9] have been recently identified in permafrost and cold seep, respectively. Given that AMGs have been demonstrated as the important toolboxes of viruses, revealing the drivers of the variation of viral AMG compositions would give us deeper insights into the ecological roles of viruses.

Recently, the environment-specific distributions of viral-encoded AMGs have been documented in the marine ecosystem [10–13]. In an analysis of the Pacific Ocean Viromes [11], AMGs involved in iron-sulfur cluster formation were only found in the iron-limited photic zone, while AMGs in the aphotic zone were related to bacterial high-pressure survival. Additionally, viral AMGs often derived from their bacterial hosts [14], suggesting that viruses infecting different microbial taxa tend to carry different AMGs, which has been reported by studies about cyanophages [15]. However, most of the previous omic-based viral researches mainly focused on the whole viral community, without distinguishing the AMGs encoded by viruses that undergo different lifestyles (lytic and lysogenic), which may hamper our fully understanding of the virus-host-environment interactions in natural ecosystems.

Viruses that adopt different lifestyles could impose distinct influences on microbes [16, 17]. After successfully invading their hosts, lytic viruses begin rapid replication and result in host death, while temperate viruses can remain latent without cell lysis until being induced [2]. More importantly, the AMGs carried by different-lifestyle viruses have been proposed to modulate microbial metabolisms in different strategies: “plunder and pillage” and “batten down the hatches” [14]. Specifically, lytic viruses could use AMGs to hijack host metabolisms and intracellular resources for progeny production when their bacterial hosts are in high density [18, 19]. While some temperate viruses could increase bacterial virulence and augment host fitness and resistance in the harsh environment via the expression of AMGs [16, 20–22]. For example, AMGs related to metabolism and membrane transportation were more abundant in lytic-enriched viromes than in temperate-enriched viromes [23]. Therefore, AMGs in different-lifestyle viruses may undergo differing selection pressures. However, whether the compositions of viral AMGs are lifestyle-dependent in natural ecosystems is still elusive.

Thus, based on these previous knowledges about viruses, we hypothesize that the variations of viral community-wide AMG compositions could be mainly driven by viral lifestyles, followed by habitats and host identities. To validate our hypothesis, using the Pearl River Estuary (PRE) as a study system, we collected 30 water (15 free-living fractions, 15 particle-attached fractions) and 14 sediment samples from 15 stations, and profiled the viruses and their AMGs utilizing metagenomic and metatranscriptomic techniques. The PRE is an ideal system for our study owing to its easy access, diverse prokaryotic microbes and habitat types [24, 25]. We also verified our findings by using the Global Ocean Viromes 2.0 (GOV 2.0) dataset.

## Materials and methods

### Sample collection and physicochemical measurement

We collected a total of 44 samples including surface waters and sediments from 15 stations along the PRE to its adjacent sea in August 2019 (Table S1). At each station, ~ 5 L surface water (~ 2–3 m depth) was sequentially filtered through a 3-μm (GSWP, Millipore, Billerica, MA, USA) and 0.22-μm membrane filter (Pall Life Sciences, Ann Arbor, MI, USA) via a peristaltic pump to collect particle-attached (PA) and free-living (FL) microbes, respectively. All the filters were frozen immediately in liquid nitrogen and stored at − 80 °C in the laboratory until DNA and RNA extraction. Additionally, the filtrates were collected in 50-mL sterile tubes and stored at − 20 °C for physicochemical measurement. The detailed procedure for collecting the surface sediments (0–20 cm; SE) has been described previously [26]. Briefly, we collected surface sediments from 14 stations using a grab sampler and froze them at − 20 ℃ for subsequent DNA extraction and physicochemical analysis.

Several water properties, including temperature, depth, dissolved oxygen, pH, salinity, turbidity, and chlorophyll a were measured in situ by conductivity-temperature-depth Rosette system (CTD). As for the nutrient concentrations, nitrate (NO_3_^−^), nitrite (NO_2_^−^), ammonia nitrogen (NH_4_^+^), phosphate (PO_4_^3−^), soluble reactive phosphate (SRP), sulfate (SO_4_^2−^), total organic carbon (TOC), total nitrogen (TN), dissolved nitrogen (DN), and total organic nitrogen (TON) were measured using standard methods as previously described [27].

### DNA and RNA extraction, and sequencing

The total DNA and RNA were extracted from each water filter (total 30 filters: 15 PA fractions, 15 FL fractions) according to the standard kit protocol (RNeasy PowerSoil Total RNA Kit, QIAGEN, Germany). DNA and RNA were separately eluted from the RNA capture column by RNeasy PowerSoil DNA Elution Kit (QIAGEN) and RNeasy PowerSoil Total RNA Kit (QIAGEN), respectively. As for the sediment samples, we extracted the total DNA using DNeasy PowerSoil Kit (QIAGEN), following the manufacturer’s protocol. The quality of all extracted DNA and RNA was detected by Agilent 2100 (Agilent, Germany). Samples that passed the quality check were sent to the Magigene Company (Guangzhou, China) for metagenomic and metatranscriptomic high-throughput sequencing on the Illumina NovaSeq 6000 PE150 platform. Finally, we obtained total 41 metagenomes (15 FLs, 12 PAs, 14 SEs) and 23 metatranscriptomes (14 FLs, 9 PAs).

The DNA samples were also used for amplifying 16S rRNA gene (V4–V5 region) using primers set: 515F (5′-GTGCCAGCMGCCGCGGTAA-3′) and 907R (5′-CCGTCAATTCMTTTRAGTTT-3′). Amplicons were barcoded, purified, and sequenced on the Illumina NovaSeq 6000 PE250 platform following the methods described previously [28]. The amplicon sequencing data were processed as previously documented [26].

### Metagenome assembly and prokaryotic genome binning

Low-quality reads and adapters were first removed from the raw metagenomic data using fastp software [29] with parameters “-n 0 -l 30 -5 -r -W 5 –cut_mean_quality 20”. Then, high-quality reads of each water sample were individually assembled using SPAdes v3.13.1 [30] with parameters “–meta -k 21,33,55,77,99,127”. Due to the high complexity of sediment microbial communities and high volume of sequencing data size (~ 60 Gb for each sample), SPAdes v3.13.1 [30] failed to handle the sediment metagenomes. Thus, we assembled the high-quality reads of each sediment sample using MEGAHIT v1.2.9 [31] with parameters “–k-min 27 –k-max 127 –k-step 20 –min-contig-len 500”. Assembly qualities (Table S2) were evaluated by QUAST v5.0.2 [32].

The detailed procedure of genome binning has been described by [26]. In brief, MetaBAT2 v2.14 [33] was used to bin the scaffolds/contigs of each sample into Metagenome-Assembled Genomes (MAGs) based on the coverage variation of contigs across samples and tetranucleotide frequencies. The qualities of MAGs were evaluated by CheckM v1.0.12 [34] and the potential contaminations were identified and removed by RefineM v0.0.25 [35] and manual curation. Medium-to-High quality MAGs (completeness > 60%, contamination < 5%) were dereplicated by dRep v2.6.2 [36] with default parameters. MAGs’ taxonomies were identified by GTDB-Tk v1.2.0 [37].

### Identification and classification of vOTUs

Viral sequences were identified from the assembled scaffolds/contigs using a combined criterion with three tools: (1) identified by VIBRANT v1.2.1 [38] with default settings; (2) classified as high-confidence viral sequences by VirSorter v2.2.3 [39] with “max_score ≥ 0.9”; (3) determined by CheckV v0.8.1 (contained at least one viral gene) [40]. For each scaffold/contig that was identified as viral sequence by all these three tools, we kept its shortest version to remove the potential host-derived contamination that was trimmed by these tools. Next, to further improve the viral genome completeness, predicted viral scaffolds/contigs whose length ≥ 3 kbp were binned into viral metagenome-assembled genomes (vMAGs) using vRhyme v1.1.0 [41] with default parameters. In total, 2252 low-contaminated viral bins were retained based on the low protein redundancy (≤ 1 redundant protein in each bin) and the reasonable scaffolds/contigs composition as previously suggested [41]. All viral bins (all scaffolds/contigs within a bin were linked) and unbinned sequences were then clustered into viral operational taxonomic units (vOTUs) using CD-HIT-EST v4.8.1 [42] with “-c 0.95 -aS 0.85” [43]. According to the previous suggested pipeline for identifying viral sequences in metagenomes [43], only vOTUs whose length ≥ 10 kb or those predicted as circular/complete genomes by VIBRANT v1.2.1 [38] and CheckV v0.8.1 [40] were retained for downstream analyses to reduce false positives.

For lifestyle prediction, temperate viruses were conservatively confirmed by prophage signals, which were identified by both VIBRANT v1.2.1 [38] and CheckV v0.8.1 [40], and manual detection of lysogeny-specific genes (i.e., integrase, recombinase, transposase, excisionase, CI/Cro repressor, and *parAB*) [44–46], while the remaining vOTUs that display no prophage signals or lysogeny-specific genes were considered as potential lytic viruses. To meet the lysogeny-specific genes detection, open reading frames (ORFs) of each viral sequence were predicted by Prodigal v2.6.3 [47] with parameters “-p meta -g 11 -f gff -q -m -c”. Then, predicted ORFs were annotated by searching against KEGG [48] using KofamScan v1.3.0 [49], eggNOG (version 5.0.0) [50] using emapper v5.0.1 [51], COG [52] and NCBI RefSeq virus databases (downloaded on 18 September 2020) [53] using DIAMOND v0.9.24 [54], and Pfam [55], VOG (release 202, http://vogdb.org/), the Prokaryotic Virus Orthologous Groups (pVOGs, release 5) [56] databases, and the HMMs profiles from CheckV v0.8.1 [40] using Hmmsearch v3.3.2 [57]. The cutoffs for all alignments were set as “e-value ≤ 10^−5^ and bit score ≥ 50”. It should be noted that, due to the incomplete assembly of viral genomes, some temperate viral sequences may contain no lysogeny-specific genes, which would lead to some misclassifications, implying that the number of true temperate viruses could be underestimated (see “Discussion” for the potential effects of this limitation).

Two methods were used to classify the vOTUs. One was the gene-sharing network analysis performed by vConTACT2 v0.9.19 [58] with the “ProkaryoticViralRefSeq94-Merged” database. The other one was gene-taxonomy-based method, where ORFs of each vOTU were searched against the NCBI RefSeq virus database using blastp v2.9.0 + [59] (e-value ≤ 10^−5^ and bit score ≥ 50) and then the taxonomy of this virus was identified by the majority-rules approach (more than half) [60] and lowest common ancestor (LCA) algorithm. Since there were no conflict taxonomic assignments between these two methods, we combined all the results from each method (Table S3).

### AMG annotation, abundance, and expression

According to the previous suggested practices for AMG curation [61], the potential AMGs were only identified within viral conserved regions, where both the start and end genes were annotated as viral hallmark or viral-like genes. Viral hallmark and viral-like genes (e-value ≤ 10^−5^ and bit score ≥ 50) were identified by searching all predicted ORFs against the pVOGs (release 5) database [56] and viral-specific HMMs profiles from CheckV v0.8.1 [40] using Hmmsearch v3.3.2 [57]. Within the conserved viral regions, nonviral ORFs were regarded as candidate AMGs and further annotated using multiple databases and methods as described in the above section “Identification and classification of vOTUs”. The functions of these ORFs were mainly identified according to KEGG annotations, and ORFs with inconsistent annotations in different databases were manually corrected to “putative protein” to maximize the annotation accuracy. Subsequently, we performed manual curation to improve the confidence in AMG identification, mainly by removing all potential illegitimate AMGs that were assigned to gene categories of DNA-related reactions, nucleotide metabolism, viral invasion (i.e., glycoside hydrolases and peptidases involved in cell wall lysis), modification of viral components (i.e., glycosyl transferases, adenylyltransferases and methyltransferases that putatively involved in viral DNA, RNA, and structural proteins modification), structural proteins, ribosomal proteins, transcriptional/translational regulators, and those unique to eukaryote as previously proposed [5, 61, 62]. Moreover, to further improve the reliability of functional annotation, predicted AMGs related to biogeochemical cycles were further filtered based on their conserved domains using the NCBI CD-search tool [63] with parameters “e-value ≤ 10^−5^ and low-complexity filtration”, and dbCAN2 server (https://bcb.unl.edu/dbCAN2/) with default settings. Finally, all obtained AMGs were classified into two classes according to the previous definition [5]: class I AMGs referred to genes involved in the “metabolism pathways” defined in the KEGG database, while genes falling into other pathways were considered as class II AMGs.

Considering that most AMGs are host-derived, to avoid the potential effects of host-derived reads on the abundances of viral-encode AMGs, we used the abundances of vOTUs to represent their AMGs’ abundances [45]. The viral abundances in each sample were calculated as FPKM (fragments per kilobase per million mapped reads) by mapping the high-quality metagenomic reads from each sample to all vOTUs using BBmap v38.70 [64] with global alignment and a minimum identity of 95%. The expression profiles of AMGs were also determined as FPKM by mapping the high-quality metatranscriptomic reads to all predicted AMGs using BBmap v38.70 [64] with a strict criterion (100% identity and 100% coverage of short reads) to minimize the potential bias caused by host-derived expressions. Next, the AMGs were further grouped into KEGG orthologs (KOs) for community-wide analyses, including the comparisons of AMG diversities, compositions, and expression profiles between different lifestyles and habitats. The KO abundances were determined by summing up the abundances of AMGs that were assigned to a given KO. Moreover, the expression level of each KO was computed as the total transcript abundances of AMGs belonging to that KO divided by its abundance.

### Construction of different datasets

To minimize the potential bias caused by the different vOTU sizes of lytic and temperate viral communities and the potential lifestyle misclassifications due to incomplete assembly, three datasets were constructed: (1) rarefied dataset: lytic viruses (*n* = 4829) in our dataset were rarified to the same vOTU number as temperate viruses (*n* = 241); (2) completeness-filtered dataset: only vOTUs whose completeness estimated to be at least 40% by CheckV v0.8.1 [40] were retained (613 lytic viruses and 120 temperate viruses); (3) rarefied completeness-filtered dataset: lytic viruses in the completeness-filtered dataset were also rarefied to the same vOTU number as temperate viruses. Furthermore, all viral populations that were larger than 10 kbp or circular in GOV 2.0 dataset [60] were downloaded, and completeness-filtered GOV 2.0 dataset was constructed following the same procedure as above.

### Viral genomic characteristics comparison

To compare the potential differences in genomic properties between lytic and temperate viruses, we calculated the gene density (ORFs number per kbp), and AMG ratio (number of AMGs/total ORFs in each vOTU) of each vOTU. Mann–Whitney *U* test was implemented to determine whether the differences are significant. It should be noted that these comparisons were conducted with the completeness-filtered dataset to reduce the potential bias caused by incomplete assembly. Additionally, 600 (81.9%) vOTUs with annotation rates ≤ 50% were also excluded before the AMG ratio comparisons to reduce the potential bias caused by poor annotations. To further validate our findings, we re-did the comparisons with the rarefied completeness-filtered dataset and completeness-filtered GOV 2.0 dataset. Similar results were obtained.

### Virus-host linkage prediction

Three methods, including homology matches, tRNAs similarity, and CRISPR spacers similarity, were used to link vOTUs to their putative prokaryotic (i.e., bacteria and archaea) hosts [65]. For host searches, in addition to the 356 medium-to-high quality MAGs recovered from our dataset, we also downloaded all representative genomes from the GTDB database (release 95) [66] and combined them as the host reference database. In the homology match detection, blastn v2.5.0 + [59] was used to align vOTUs to the host reference database with parameters “identity ≥ 70, query coverage ≥ 75, e-value ≤ 10^−3^, bit score ≥ 50” as previously suggested [67]. As for the tRNAs similarity method, the tRNAs in vOTUs were identified by tRNAScan-SE v1.23 [68] using the bacterial and archaeal mode, and then, the obtained tRNAs were queried against the host reference database using blastn v2.5.0 + [59] with parameters “identity = 100, coverage = 100”. As for the CRISPR spacers similarity method, CRISPRCasFinder [69] with default parameters was used to determine and extract the spacer sequences from the host reference database. Spacers were then queried against all vOTUs by blastn v2.5.0 + [59] with parameters “word size = 16, coverage = 100, mismatch ≤ 3, e-value ≤ 10^−6^” [70]. Finally, the hosts predicted by any of the methods were combined as the final potential hosts of viruses. All predicted virus-host linkages are listed in Table S4.

### Statistical analyses

All statistical analyses were carried out in R software (version 4.0.3). Non-metric multidimensional scaling (NMDS) analysis was conducted to cluster the viral communities and AMG profiles (KOs’ relative abundance) of different samples based on the Bray–Curtis distance. Their significant differences between different lifestyles and habitats were further verified using “Adonis” function in *vegan* package with 999 permutations. The Mantel test (999 permutations) was used to calculate the pairwise correlations among the Bray–Curtis dissimilarities of viral communities and prokaryotic communities, and the Euclidean distance of environmental factors. In addition, Spearman correlations were calculated to reveal the relationships between vOTU numbers and environmental factors. The significance of differences in viral relative abundance among three habitats (i.e., water, particle, and sediment) was identified by analysis of variance (ANOVA), while the differences in the active AMG (KO) numbers between two lifestyles and two water fractions (i.e., FL and PA) were determined using Mann–Whitney *U* test.

Comparisons of the community-wide AMG diversities were performed at the KO level. Briefly, the Richness and Shannon indexes of the KOs in each sample were determined using the “diversity” function in *vegan* package, and then compared using Mann–Whitney *U* test. To identify the enriched AMG functions in each lifestyle, we clustered the AMGs into functional pathways based on the KEGG database, calculated the frequencies of viruses that encoded AMGs belonging to specific functional pathway in different viral communities (lytic and temperate), and then verified their differences using Fisher’s exact test. Same procedures were also performed using rarefied dataset, completeness-filtered dataset, rarefied completeness-filtered dataset, and GOV 2.0 dataset.

To explore whether the functional profiles of viral-encoded AMGs were distinct among three habitats, the differences of KOs’ relative abundance among habitats were evaluated using Kruskal–Wallis test. Enriched KOs (*P* < 0.05) in each habitat were then grouped into functional pathways. Additionally, to compare the AMG functional profiles among different host taxa, viruses (lytic, *n* = 167; temperate, *n* = 30) that linked with only one predicted host order were selected and grouped by their host orders. Within each viral group, the occurred frequency of viruses that carried AMGs belonging to a given pathway was calculated and further compared with other groups using Fisher’s exact test.

## Results

### Overview of viral communities and their environmental drivers

**Fig. 1:**
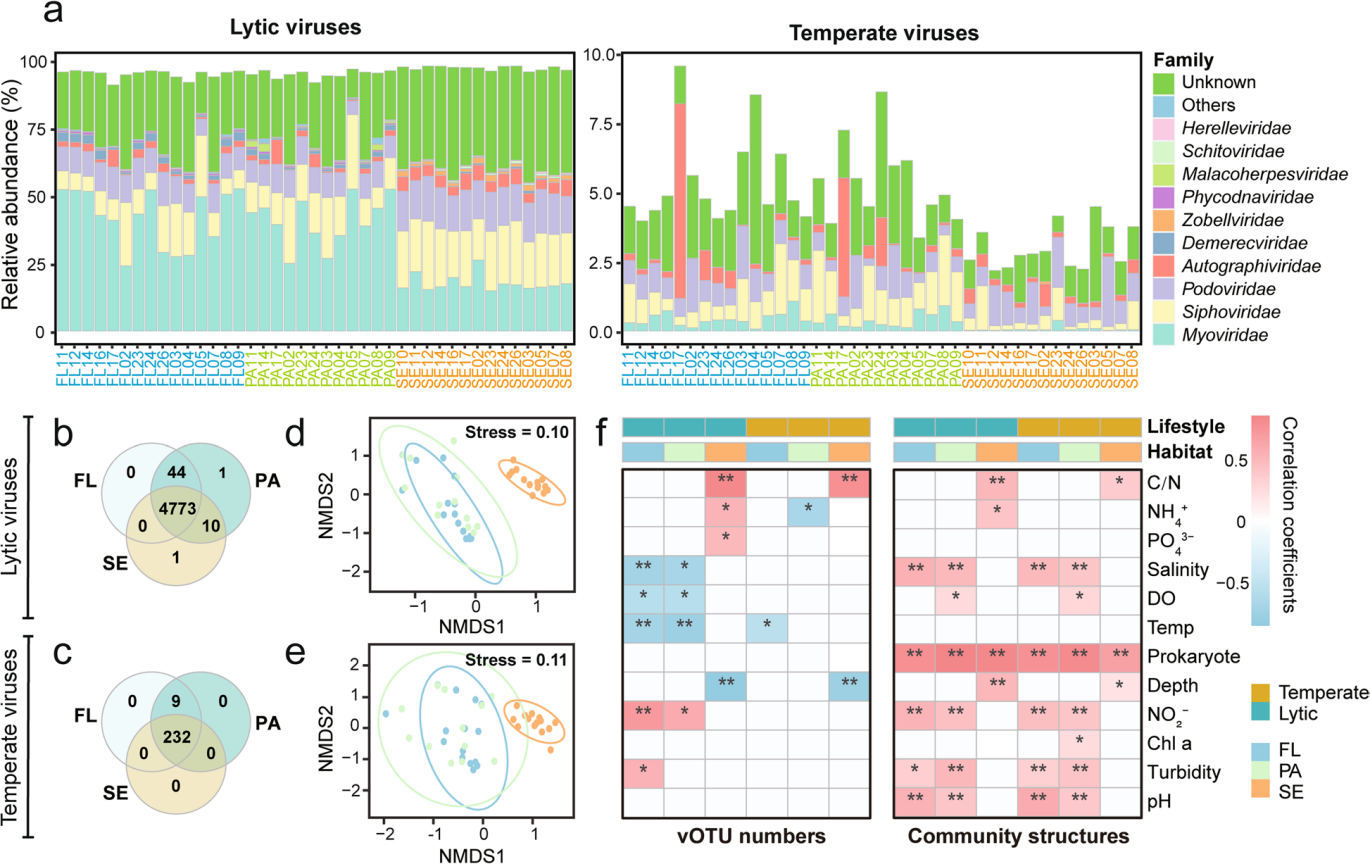
Viral community structures and their environmental drivers.** a** Community compositions (family level) of lytic and temperate viruses in all samples. **b, c** Venn diagrams display the number of unique and shared (lytic and temperate) vOTUs in the three habitats. **d, e** NMDS of (lytic and temperate) viral communities based on Bray–Curtis dissimilarity. **f** The left heatmap shows the Spearman’s correlations between (lytic and temperate) vOTU numbers and environmental factors (Prokaryote: prokaryotic richness). The right heatmap shows the Mantel correlations between Bray–Curtis distance of viral community structures and Euclidean distance of each environmental factor (Prokaryote: Bray–Curtis distance of prokaryotic communities). Color bars on the top of the heatmaps indicate the viral lifestyles and habitats, respectively. The number of asterisks denotes the statistical significance level (* *P* < 0.05 and ** *P* < 0.01). C/N, ratio of carbon to nitrogen; Temp, temperature; Chl a, chlorophyll a; FL, free-living; PA, particle-attached; SE, sediment

In total, 5070 vOTUs (≥ 10 kbp or circular/complete) were recruited from our dataset based on the in silico viral prediction (Table S3). Of these vOTUs, 241 were identified as temperate viruses, while the remaining 4829 vOTUs without lysogeny-specific genes or prophage signals were inferred as potential lytic viruses. As expected, the total relative abundance of lytic viruses, accounting for 91 ~ 98% of the whole viral communities, was significantly higher than that of temperate viruses (Fig. 1a). Additionally, the viral family-level community structures differed between these two lifestyles, even though they were both dominated by the order *Caudovirales* (Fig. 1a and Table S3). Lytic viruses had higher taxonomic diversity and the *Myoviridae* was the most abundant lytic virus in both waters and sediments, while the family *Siphoviridae* and *Podoviridae* dominated the temperate viruses. Moreover, most of the lytic (98.8%) and temperate (96.2%) viruses were shared by the three habitats (i.e., water, particle, and sediment; Fig. 1b, c).

In each habitat, we found that the number of vOTUs was greater near shore (lytic: north shore; temperate: west shore for FL and PA, north shore for SE) and decreased with increasing offshore distance (Figs. S1a, b), which may be driven by the changes of salinity, dissolved oxygen, temperature, nutrient concentration, and turbidity (Fig. 1f). When compared among three habitats, the relative abundance of lytic viruses was significantly higher in sediment than in water (Fig. S1c), whereas temperate viruses showed opposite pattern (Fig. S1d). The ordination plot further confirmed the significant difference between viral communities of water and sediment (Adonis: lytic, *R*^2^ = 0.30, *P* = 0.001; temperate, *R*^2^ = 0.26, *P* = 0.001; Fig. 1d, e). Furthermore, we found that the viral community dissimilarities were positively correlated with the differences in prokaryotic communities (Mantel’s *r* = 0.7 ~ 0.9, *P* < 0.01; Fig. 1f). Moreover, several environmental factors, such as water nitrite, pH, salinity, and sediment C/N ratio, also showed strong correlations with the changes of lytic and temperate viral communities (Mantel’s *r* = 0.40 ~ 0.61, *P* < 0.01; Fig. 1f).

### Lifestyle-dependent viral genomic properties and AMG compositions

**Fig. 2:**
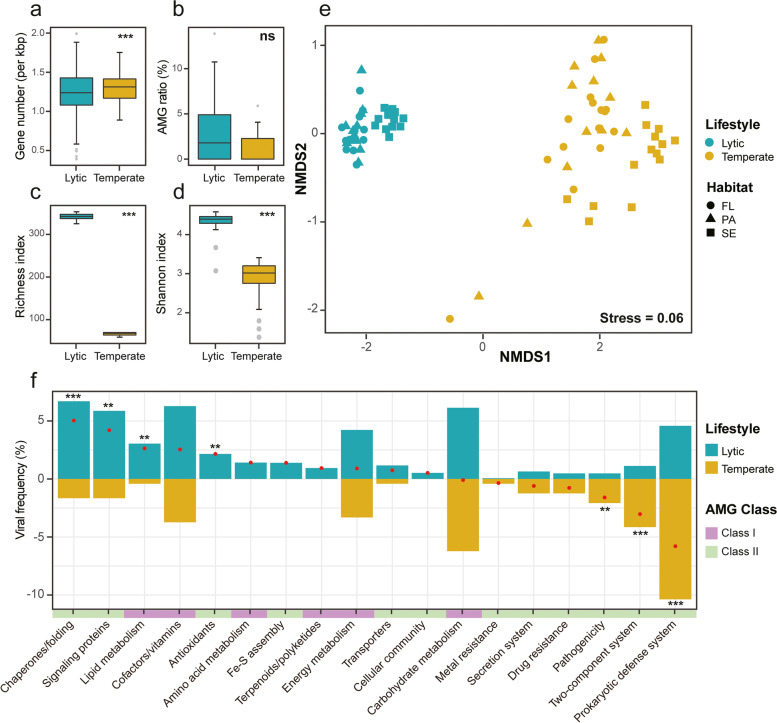
Comparisons of viral genomic properties and compositions of AMG functions. Gene densities (**a**) and AMG ratios (**b**) of high-genome-completeness vOTUs, and AMG diversities (**c, d**) of lytic and temperate viral communities. The significance of the differences was determined by Mann–Whitney *U* test (*** *P* < 0.001). **e** NMDS of AMG compositions based on Bray–Curtis dissimilarity of KOs’ relative abundance in different samples. FL, free-living; PA, particle-attached; SE, sediment. **f** Occurred frequency of viruses carrying specific function in the whole lytic (blue bars) or temperate (yellow bars) viral communities. Red dots represent the differences of frequency between two lifestyles (lytic minus temperate). The asterisks on top of the bars indicate the statistical significance level (Fisher’s test, ** *P* < 0.01, *** *P* < 0.001)

To reduce the potential bias caused by the incomplete assembly, vOTUs with high genome completeness (completeness ≥ 40%; lytic, *n* = 613; temperate, *n* = 120) were used to compare the gene densities and AMG ratios between different lifestyles. We found that the gene densities were significantly higher in temperate viruses than in their lytic counterparts (Fig. 2a and Table S3), while the AMG ratios showed no significant difference between lifestyles (Fig. 2b). After rarefying the lytic viruses, consistent results were observed (Fig. S2).

In our dataset, 4207 and 155 AMGs belonging to 344 and 69 KEGG orthologs (KOs) were identified in lytic and temperate viral communities, respectively (Tables S5 and S6). At the KO level, we found that the functional diversity of AMGs was greater in lytic viral communities than temperate viral communities (Fig. 2c, d). Moreover, the compositions of AMGs were significantly different between viral lifestyles, irrespective of what habitats the viruses came from (Adonis, *R*^2^ = 0.48, *P* = 0.001; Fig. 2e). Specifically, we found that AMGs involved in chaperones and folding, signaling proteins (mainly P-starvation inducible protein PhoH), lipid metabolism, and anti-oxidation were significantly enriched in the lytic viral community (Fig. 2f and Table S7), while AMGs associated with defense system, two-component system, and pathogenicity were significantly enriched in the temperate viral community (Fig. 2f and Table S7). Furthermore, AMGs involved in amino acid and terpenoids/polyketides metabolisms, anti-oxidation, Fe-S cluster assembly, and cellular community were only found in the lytic viral community (Table S7). Analogous AMG distributions were also observed in the rarefied dataset, completeness-filtered dataset, and rarefied completeness-filtered dataset (Figs. S3 and S4). Importantly, we further verified these findings using the GOV 2.0 dataset and also obtained similar results (Fig. S5), except the comparison of AMG ratio.

**Fig. 3:**
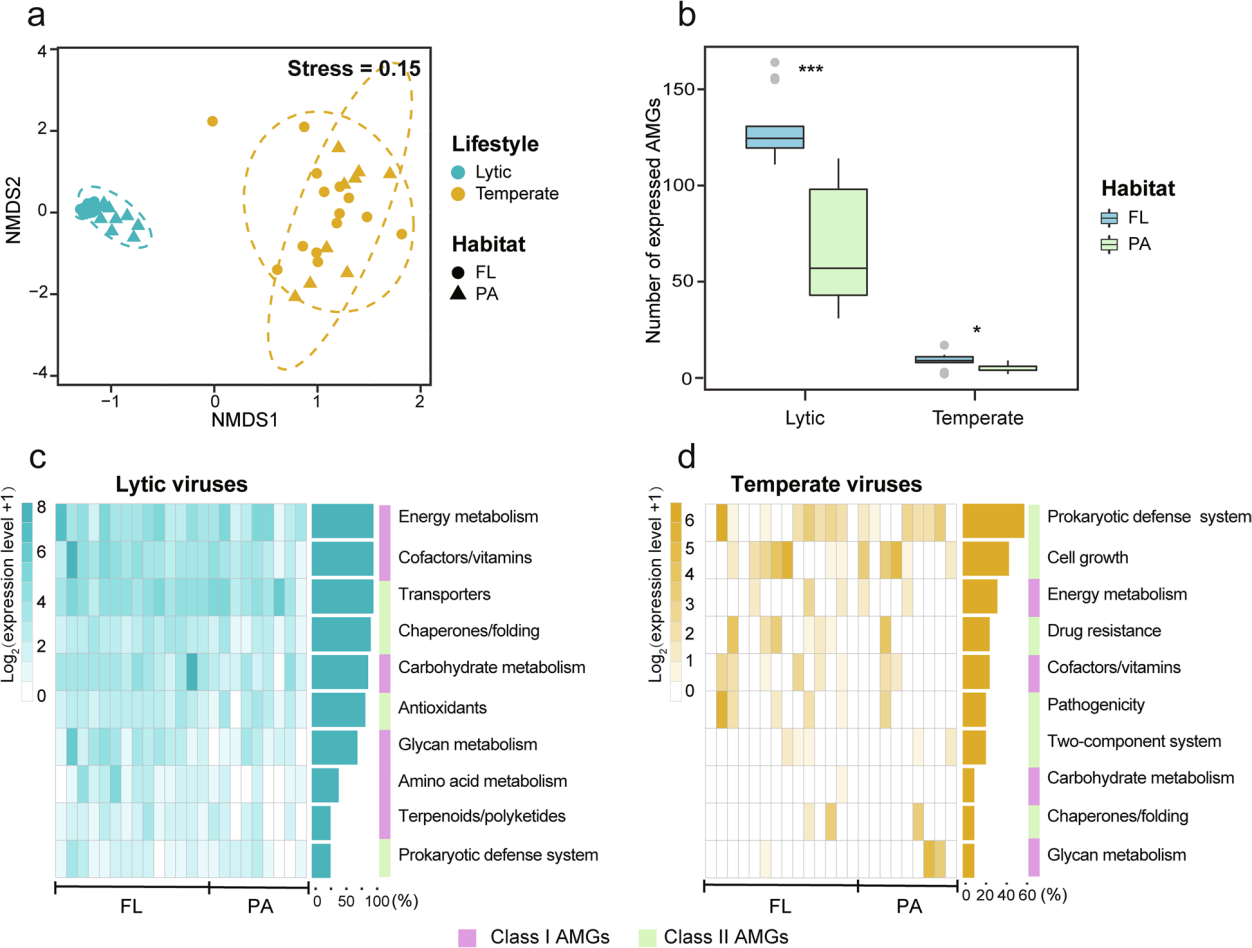
Expression profiles of lytic and temperate viral communities.** a** NMDS of the expression profiles of viral AMGs at KO level in the FL (free-living) and PA (particle-attached) samples. The expression level of each KO was calculated as the expression abundance (FPKM) divided by gene abundance (FPKM). **b** Numbers of active AMGs (KOs) across different lifestyles and habitats. The significant differences between the FL and PA samples were determined by Mann–Whitney *U* test (* *P* < 0.05, *** *P* < 0.001). Heatmaps of the highly expressed functional pathways in the lytic (**c**) and temperate (**d**) viral communities across different samples. The sidebar lengths represent the relative frequencies of samples, in which the expression level of the given AMG function was in the top ten (lytic) or top five (temperate). The right color bars showed the AMGs’ functional classes

In parallel with the AMG compositions, the expression profiles of AMGs (KO level) were also apparently separated between lytic and temperate viral communities (Fig. 3a; Tables S8 and S9). Specifically, the number of active AMG functions (KOs) in lytic viral communities was significantly greater than those in temperate viral communities (Mann–Whitney *U* test, *P* < 0.001; Fig. 3b). In lytic viral community, AMGs associated with energy metabolism, cofactor/vitamin metabolism (mainly folate biosynthesis), transportation, and chaperones and folding were most frequent and active (Fig. 3c), whereas temperate viruses typically expressed AMGs that were involved in defense system, cell growth regulation, and stress responses (including antibiotic resistance and two-component system; Fig. 3d). Similar results were obtained using the rarefied dataset (Fig. S6). Furthermore, we also found that temperate viruses tended to encode and highly express class II AMGs during infections (Figs. 2f and 3d).

### Habitat-dependent viral AMG compositions

**Fig. 4:**
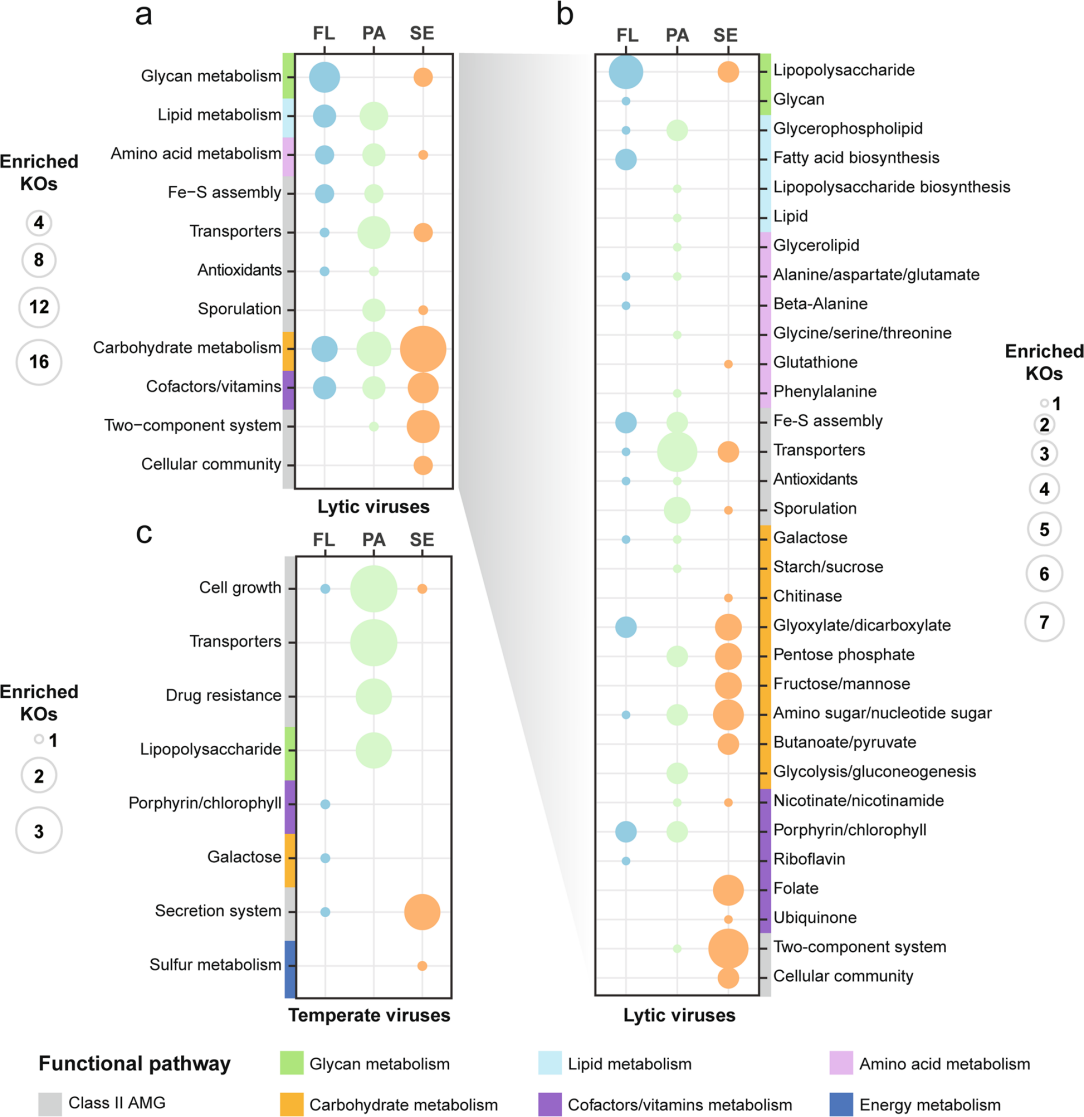
The compositions of viral community-wide AMGs across three habitats. Bubble sizes show the number of enriched AMG functions (KOs) belonging to specific functional pathway in each habitat. The enriched KOs were determined by the Kruskal–Wallis test (*P* < 0.05) based on their relative abundance in the lytic (**a, b**) and temperate (**c**) viral communities among three habitats. FL, free-living; PA, particle-attached; SE, sediment

When focusing on each lifestyle, we observed a significant partitioning of AMG compositions between water and sediment (Adonis: lytic, *R*^2^ = 0.42, *P* = 0.001; temperate, *R*^2^ = 0.23, *P* = 0.001; Fig. 2e), consistent with the viral community structures (Fig. 1d, e). Specifically, lytic viruses in water fractions (including FL and PA) enriched more AMGs involved in transportation and multiple core metabolisms, in particular, glycan, lipid, and amino acid metabolisms (Fig. 4a), whereas lytic viruses in sediment encoded a higher proportion of AMGs related to carbohydrate (especially chitin, fructose/mannose, butanoate, and pyruvate) and cofactor/vitamin (i.e., folate) metabolisms, and two-component system (Fig. 4a, b and Table S10). As for the temperate viruses, AMGs related to cell growth regulation, transportation, antibiotic resistance, and lipopolysaccharide metabolism were typically enriched in water fractions, while sedimental temperate viruses tended to encode AMGs involved in secretion system (i.e., type VIII secretion system), as well those related to sulfur metabolism (Fig. 4c and Table S10).

Although the AMG compositions of FL and PA fractions did not show significant differences (Adonis: lytic, *R*^2^ = 0.02, *P* = 0.79; temperate, *R*^2^ = 0.05, *P* = 0.25; Fig. 2e), the AMG expression patterns of lytic viruses in these two fractions were markedly different (Adonis, *R*^2^ = 0.09, *P* = 0.005; Fig. 3a). Lytic viral AMGs involved in nutrient metabolism, Fe-S assembly, and stress response showed significantly higher expression in FL fraction than in the PA fraction (Table S11). As for temperate viruses, the AMG expressions showed no significant difference between two water fractions (Adonis, *R*^2^ = 0.03, *P* = 0.92; Fig. 3a and Table S11). Moreover, viruses (both lytic and temperate) in FL fractions exhibited more active AMGs (KOs) than those in PA fractions (Fig. 3b).

### Host-specific viral AMG compositions

Utilizing multiple methods (see “Materials and methods”), we finally linked 204 lytic and 35 temperate viruses to 26 and 7 predicted prokaryotic host phyla, respectively (Table S4), with most frequently predicted hosts belonging to Proteobacteria, Bacteroidota, Cyanobacteria, and Actinobacteriota (Fig. S7). Moreover, while most of the viruses had a narrow host range (one host phylum), approximately 10.2% (lytic) and 5.7% (temperate) host-annotated viruses exhibited a broader host spectrum across phyla (≥ 2 phyla; Fig. S8). Interestingly, three lytic viruses were predicted to infect both bacteria and archaea (e.g., Cyanobacteria and Nanoarchaeota), suggesting that some viruses could infect hosts across domains (Fig. S8a). However, these debatable cross-phyla/cross-domain host predictions were predicted solely based on tRNA homology, and further evidences are needed. 

**Fig. 5:**
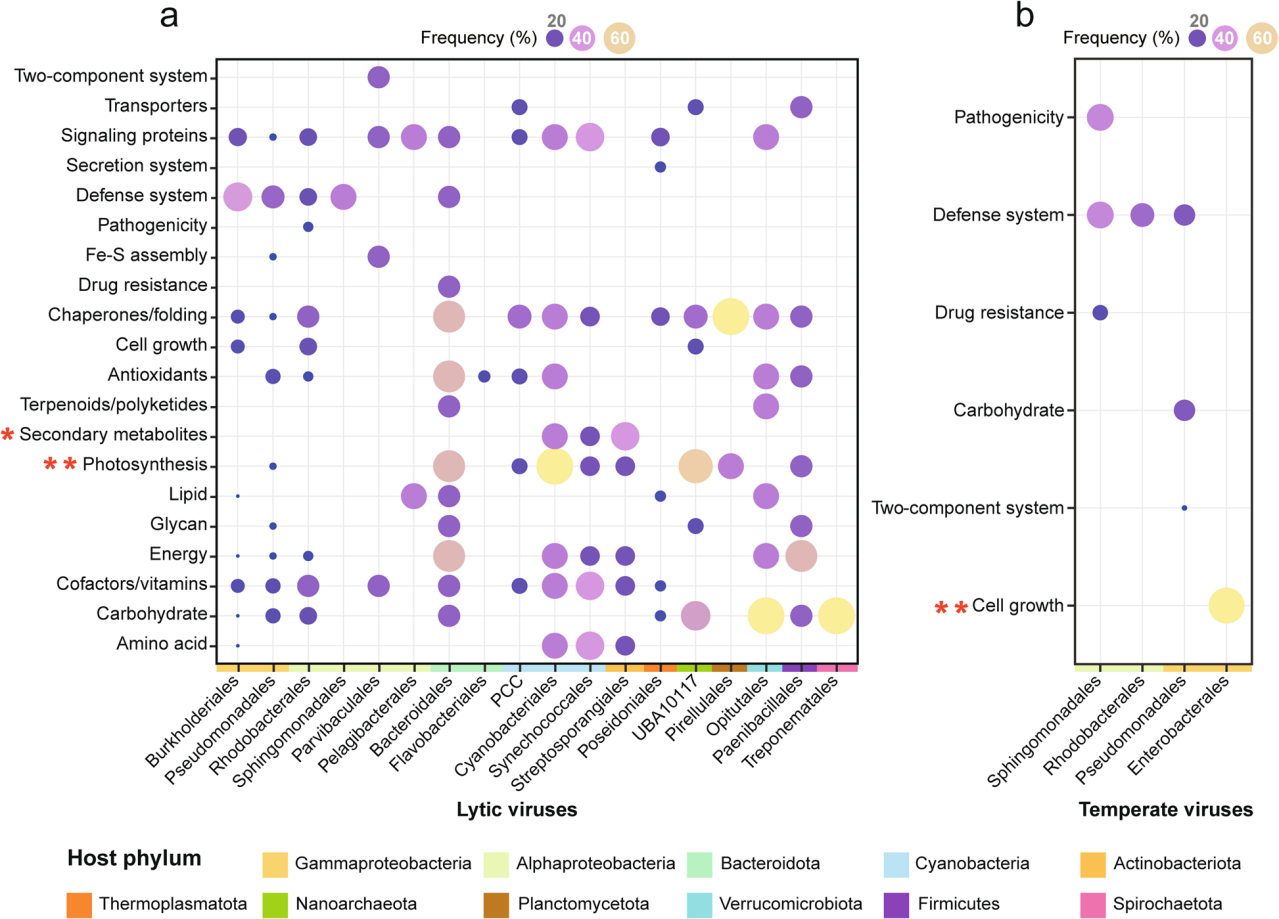
The compositions of viral community-wide AMGs across different host orders. Bubble sizes show the occurred frequency (%) of viruses that encode AMGs belonging to specific functional pathway, among all lytic (**a**) or temperate (**b**) viruses that could infect the given host order. The statistical significance was determined by Fisher’s exact test (* *P* < 0.05, ** *P* < 0.01)

Given the fact that viral community-wide AMG compositions showed strong associations with the prokaryotic community structures (Mantel’s: lytic, *r* = 0.73, *P* = 0.001; temperate, *r* = 0.38, *P* = 0.001), we further explored whether the viral AMG compositions are host-specific. We found that although AMGs involved in cofactor/vitamin metabolisms, chaperones and folding, and signaling proteins were widely occurred in all lytic viruses, their occurred frequencies differed among viruses with different hosts (Fig. 5a and Table S12). Lytic viruses infecting Cyanobacteria typically encoded AMGs related to photosynthesis, as well genes involved in amino acid and cofactor/vitamin metabolisms. Notably, some photosynthesis AMGs were also found in viruses infecting non-photosynthetic prokaryotes, especially Bacteroidota and Nanoarchaeota (Fig. 5a). Moreover, secondary metabolite-related AMGs were more commonly found in the genomes of viruses that infect *Streptosporangiales* (Actinobacteriota), whereas AMGs involved in carbohydrate metabolism were prevalent in viruses linked to *Opitutales* (Verrucomicrobiota) and *Treponematales* (Spirochaetota). Interestingly, viruses infecting Nanoarchaeaeota harbored diverse AMGs (Table S12), suggesting that viruses probably can increase the fitness of DPANN archaea by expanding their metabolic capabilities via AMGs. Compared with the lytic viruses, temperate viral AMG compositions displayed a stronger host specificity (Fig. 5b and Table S13). Specifically, cell growth-related AMGs were commonly found in the viruses that infected *Enterobacterales* (Gammaproteobacteria). While temperate viruses infecting *Sphingomonadales* (Alphaproteobacteria) harbored both pathogenic and antibiotic-resistant genes, which were not found in other host-specific temperate viruses in our dataset.

### Abundant biogeochemical-cycle related AMGs in lytic viruses

**Fig. 6:**
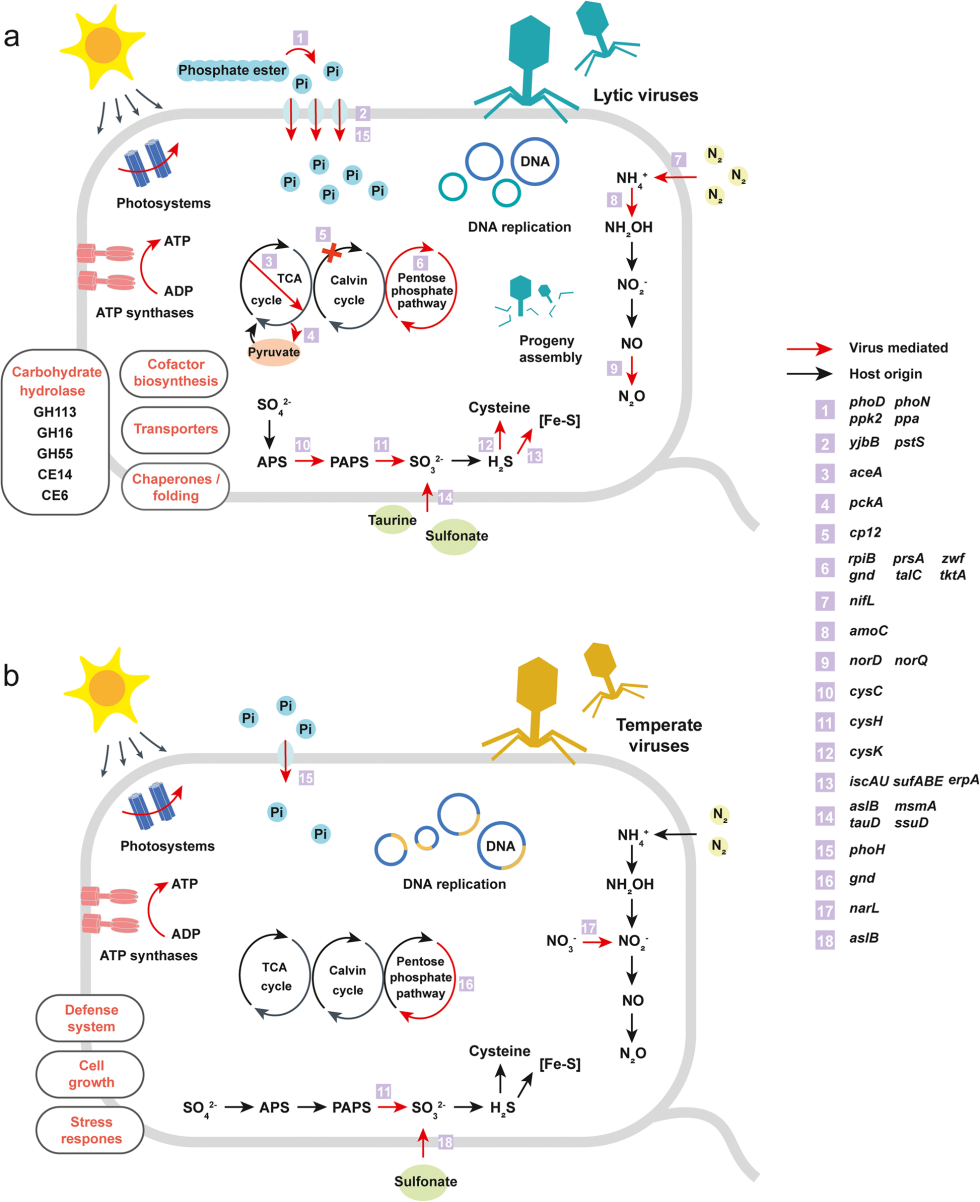
Conceptual diagrams depicting the virus-host interactions via AMGs. The diagrams show how lytic (**a**) and temperate (**b**) viruses may modulate host metabolism in the PRE. Red arrows indicate the reactions that viruses may actively participate in, confirmed by their AMGs’ expression levels. **a** Upon successful infections, lytic viruses could strongly shape their host metabolisms and the biogeochemical cycles by expressing AMGs involved in organic matter degradation (i.e., carbohydrate, phosphate ester, and organosulfur), nutrient uptake, and transformation, which in turn redirect the energy and materials toward viral progeny assembly. **b** For temperate viruses, they typically express AMGs that could enhance prokaryotic defense system and regulate cell growth for host survival. They also tend to augment host environmental tolerance by expressing AMGs involved in antibiotic resistance and two-component systems

In our dataset, numerous active AMGs involved in carbon, nitrogen, phosphorus, and sulfur cycling were identified (Fig. S9 and Table S14), and they showed different expression patterns within different lifestyles (Fig. S10). In lytic viruses, the most active AMGs were involved in nitrogen metabolism, including ammonia monooxygenase genes (*amoC*), nitric oxide reductase genes (*norQ*, *norD*), nitrogen fixation genes (*nifL*), and ammonia assimilation genes (*asnB*, *glnA*). Besides, lytic viruses were also active in the degradation of organic matters, including carbohydrate (GH16, GH55, GH113, CE6, and CE14), phosphate ester (*phoD*, *phoN*), inorganic phosphate (*ppa*, *ppk2*), and organosulfur compound (*aslB*, *msmA*, *tauD*, *ssuD*). Furthermore, AMGs involved in nutrient transportation, assimilation, and transformation were also highly expressed, indicating that lytic viruses could enhance the nutrient utilization of their hosts to benefit viral replication (Fig. 6a).

Compared with lytic viruses, temperate viruses expressed fewer types of biogeochemical-cycle related AMGs during infection (Figs. 6b and S10b). Of these AMGs, P-starvation response regulators (*phoH*) were the highest expressed, followed by nitrate-nitrite response regulators (*narL*). Considering that metagenomic technology could not avoid some incorrect lifestyle classifications, we might, somewhat, underestimate the ecological roles of temperate viruses in biogeochemical cycling.

## Discussion

In this study, using the PRE as a model system, we systematically examined the drivers of viral community-wide AMG compositions by utilizing multi-omics techniques. We found that viral lifestyles were the most important drivers, followed by habitats and host identities.

Viruses within two lifestyles were appreciably distinguished by their gene densities, AMG diversities, compositions, and expressions (Figs. 2 and 3). These differences are most likely attributed to their disparate evolutionary backgrounds. Unlike lytic viruses, temperate viruses often integrate themselves into their host genomes as prophages and replicate with their hosts [16, 17]; therefore, a smaller genome size [46, 71], which could alleviate host metabolic burden [72], should be better for the survival of the temperate viruses. In this context, higher gene density in temperate viruses (Fig. 2a) could be a fine strategy to maximize their genetic information in a limited genome length. Contrasting to the gene density, we found that the AMG diversity was greater in lytic viral community. This finding was also consistently identified in other datasets (Figs. S3 and S5c, d), indicating that the greater AMG diversity in the lytic viral community should be a biological phenomenon. Moreover, previous studies have proposed that horizontal gene transfer (HGT) was a crucial driver of viral evolution [1, 46] and its frequency varied with viral lifestyles [46, 71]. In particular, temperate viruses often experience higher HGT frequency than lytic viruses. Although more frequent HGT could facilitate the functional diversification in viral genomes [71], it also might homogenize the AMG composition of the whole temperate viral community, which in turn, results in lower AMG diversity. Additionally, lytic viruses were able to infect a broader range of host species than temperate viruses [71]. With a broader host range and higher infection frequency in their life histories, lytic viruses likely get access to a larger AMG pool and thus tend to own higher AMG diversity. Considering the key roles of viral AMGs in modulating hosts’ metabolisms, higher AMG diversity in lytic viruses suggests their more versatile ways in interacting with their prokaryotic hosts.

Furthermore, different AMG compositions in lytic and temperate viral communities highlighted the distinct virus-host interactions of different-lifestyle viruses. While the ecological roles of specific viruses have been explored using model systems by previous studies [18, 19], here, we obtained all viral-encoded AMGs from the whole viral community via omics techniques, allowing for a more comprehensive exploration at the community scale in natural conditions. We found that lytic viruses typically enriched AMGs involved in chaperones and folding catalysts (e.g., heat shock proteins IbpA), phosphate starvation response, lipid metabolism (e.g., glycerophospholipid metabolism), and antioxidants (Fig. 2f). As these AMGs all participate in the key processes for supporting cellular energy and materials [4, 73, 74], our findings indicate that lytic viruses could hijack their hosts’ metabolisms to accelerate the biosynthesis of viral components, consistent with previous studies based on model systems [19, 75, 76]. Conversely, temperate viruses tended to enhance host survivability via AMGs (Fig. 2f), which agreed with the previous findings in isolated temperate viruses [20]. For instance, temperate viruses enriched AMGs involved in prokaryotic defense system (e.g., restriction-modification system), implying their roles in protecting their host from superinfection [77]. AMGs related to pathogenicity and antibiotic resistance (mainly beta-lactam resistance) were also significantly enriched in temperate viruses. These AMGs, which have been previously discovered in marine and freshwater viromes [3, 78], could potentially contribute to extending the ecological niches of their hosts [17]. Furthermore, temperate viruses might enhance their host abilities for sensing and responding to environmental cues via activating host two-component systems [79], particularly under fluctuating conditions. Overall, our results strongly suggest that lytic viruses tend to encode AMGs to redirect host core metabolisms for progeny production, whereas temperate viruses are apt to encode AMGs that shape host physiological states for mutualism. Additionally, it should be noted that some potential misclassifications of temperate viruses were unavoidable using current methods, but these misclassifications should not result in the aforementioned findings, especially with the fact that analogous functional distributions of AMGs were also observed in other datasets (Figs. S4 and S5f).

When focusing on each lifestyle, we found that the AMGs were, to some extent, host-specific. For example, AMGs involved in photosynthesis (that is, photosystem II and plastocyanin) and amino acid metabolism were typically found in lytic viruses infecting Cyanobacteria (Fig. 5a). Viruses with these AMGs could boost host photosynthesis and material biosynthesis [18], which in turn facilitate viral replication. Moreover, carbon metabolism-related AMGs were enriched in lytic viruses that infect known carbohydrate degraders, including Bacteroidota, Verrucomicrobiota, Firmicutes, and Spirochaetota [80]. Viruses carrying these genes might enhance their hosts’ carbon utilization, as well, redirect the carbon fluxes toward viral assembly [12]. Interestingly, AMGs involved in carbon metabolism (mainly pentose phosphate pathway) were also abundant in lytic viruses infecting Nanoarchaeota. As most DPANN archaea lack complete pentose phosphate pathway [81, 82], viruses carrying these AMGs could presumably compensate their hosts’ incomplete pathway to enable nucleotide and nucleic acid synthesis for viral replication. In the temperate viral communities, viruses that infect *Enterobacterales* typically carried cell growth regulation AMGs (i.e., ATP-dependent Clp proteases; Fig. 5b), which were foundations for virulence and stress tolerance in pathogenic bacteria [83]. Collectively, such host-specific AMG compositions provide powerful evidence for the co-evolution of viruses and their hosts under natural environments.

The compositions of viral community-wide AMGs were also strongly shaped by the environmental conditions (Fig. 4). Considering the strong co-evolution relationships between viruses and hosts, we suspected that the habitat-dependent AMG compositions may mirror the adaptation of their prokaryotic hosts to the environments. Specifically, viruses living in water enriched AMGs involved in nutrient metabolism (lytic viruses), transportation, and cell growth regulation (temperate viruses), which could enhance their hosts’ nutrient uptake and metabolism ability, as well environmental tolerance. These AMGs may confer fitness advantages for both host and viruses under the relatively nutrient-limited conditions (comparing with sediment) and strong ultraviolet stress of water. By contrast, the PRE sediments are rich in nutrients (especially the animal and plant-derived polysaccharides and organosulfur compounds [84, 85]) and nourish many prokaryotic species and individuals [86]. To better adapt to this sedimentary environment, viruses would likely enrich more AMGs associated with carbon (e.g., chitin, fructose/mannose, butanoate, and pyruvate; lytic viruses) and sulfur metabolisms (temperate viruses) to increase their host competitiveness for resources [6, 87], and then facilitate viral progeny production. Abundant carbon and sulfur metabolic AMGs were also found in mangrove and marine sediments [9, 87, 88]. Meanwhile, temperate viruses living in sediment enriched type VIII secretion system-related AMGs (i.e., curli secretion-assembly genes *csgGF*), which play key roles in promoting bacterial biofilm formation and modulating host community behaviors [89, 90]. Therefore, temperate viruses carrying these AMGs may confer population-level benefits for their hosts in the severe interspecies competition [22, 91].

Recently, utilizing omics techniques, several studies have demonstrated that viruses could play important roles in biogeochemical cycling [6, 92, 93]. Here, we further found that the roles of viruses in biogeochemical cycling could also be lifestyle-dependent, with lytic viruses in the PRE expressing more AMGs involved in nutrient degradation, mineralization, transportation, assimilation, and transformation than temperate viruses (Figs. 6 and S10). Moreover, viruses within different lifestyles differentially mediated microbial-driven nitrogen cycling via AMGs. In the nitrogen-polluted PRE, lytic viruses actively expressed *amoC* genes (Fig. S10), which may boost microbial ammonia oxidation. Similar phenomena were also found in the Gulf of Mexico and freshwater lakes [8, 94]. Furthermore, nitric oxide reductases (NorQ and NorD), the vital membrane proteins required for reducing NO to N_2_O [95], showed high transcriptional levels in lytic viruses. Given the heavy nitrogen pollution [26, 96], nitrification and denitrification were very active in the PRE and these processes could generate NO that is harmful to microbes [97]. Thus, the viral-encoded nitric oxide reductases may help their hosts release NO stress, but may also result in more emission of N_2_O (a potent greenhouse gas). Intriguingly, the expression level of *norD* genes was negatively related to the oxygen concentration (Fig. S10a), suggesting that these genes may also be a selective advantage for safeguarding host energy production in low-redox environments [7]. Moreover, lytic viruses can also potentially regulate the activity of nitrogenase in their hosts by expressing *nifL* genes [98]. Whereas for temperate viruses, they carried and expressed *narL* genes, which are crucial response regulators for extracellular nitrate/nitrite [99, 100], and could subsequently activate host nitrate reductases. In addition to the nitrogen cycling, we also found many active AMGs (especially in lytic viruses) that are associated with the rate-limiting steps in carbon, phosphorus, and sulfur cycles (Fig. 6a). Such abundant biogeochemical-related AMGs in the lytic viral community may promote microbial nutrient uptake and utilization in the PRE, which in turn boost viral replication. More importantly, such abundant active biogeochemical-cycle related AMGs imply that lytic viruses could also directly affect the biogeochemical cycles via modulating prokaryotic metabolisms, not just by lysing cells (namely “viral shunt”) [76].

To the best of our knowledge, our findings provide a novel insight into the lifestyle-dependent AMG compositions at the viral community scale. As AMGs are the important bridges between viruses and hosts, these findings have practical implications for regulating the bacterial-mediated bioprocess [28, 101, 102]. However, several limitations should be noted. First, in this study, the lifestyles of viruses were inferred based on the lysogeny-specific genes and prophage signals without any experimental verification. Due to the limitations of metagenome assembly, some viral sequences were not complete, which might lead to the misclassifications of some temperate viruses. But some potential misclassifications should not change our conclusions, given the fact that after rarefying the lytic vOTUs, removing low-completeness vOTUs and using the GOV 2.0 dataset, we still obtained similar results (Figs. S2, S3, S4, S5 and S6). Second, due to the limitation of reference database, many AMGs’ functions were unknown and were removed from our analyses, which may, to some extent, hamper our comprehensive view of the viral functions. Thus, lots of work is still needed before we can fully understand the ecological roles of diverse viruses in natural ecosystems.

## Conclusion

Viral-encoded AMGs act as key agents in modulating microbial activities and biogeochemical cycles, yet information regarding the drivers that affect the AMG distributions in natural ecosystems is still limited. Utilizing omics’ techniques, our comprehensive analysis of the viral AMG diversities, compositions, and expression profiles at a community-wide scale successfully revealed that viral lifestyles were the most important drivers, followed by habitats and host identities. Specifically, lytic viruses featured remarkably high AMG diversity and enriched AMGs for boosting host metabolisms, which benefit viral replication. Conversely, temperate viruses tended to encode more AMGs related to microbial physiology regulations, and could subsequently facilitate virus-host mutualism. Moreover, lytic viruses expressed more AMGs related to nutrient metabolism and modulated different steps in the nitrogen cycle when compared with temperate viruses, highlighting their distinct roles in biogeochemical cycling. These distinctions might be tightly associated with the different evolutionary histories of lytic and lysogenic lifestyles. Additionally, viruses within each lifestyle also exhibited habitat-dependent and host-specific AMG compositions. Overall, these findings largely advance our understanding of the complex interactions among viruses, hosts, and the environments. Furthermore, further improvement of lifestyle classification, gene annotation, and global research will provide more insights into the ecological consequences of the viral AMG differentiations among different lifestyles, habitats, and hosts.

## Supplementary Information

**Additional file 1: Fig. S1.** Geographic distributions of viruses. **Fig. S2.** Comparisons of the genomic properties between lytic and temperate viruses in the rarefied completeness-filtered dataset. **Fig. S3.** Comparisons of the functional diversities and compositions of AMGs between lytic and temperate viruses in three datasets. **Fig. S4.** Lifestyle-dependent AMG compositions in three datasets. **Fig. S5.** Comparisons of the viral genomic properties and compositions of AMG functions based on GOV 2.0 dataset. **Fig. S6.** Expression profiles of lytic and temperate viral communities in the rarefied dataset. **Fig. S7.** Virus-host linkages at phylum level. **Fig. S8.** Host ranges of the lytic and temperate viruses in the PRE. **Fig. S9.** Biogeochemical-cycle related AMGs in the PRE viruses. **Fig. S10.** Expression profiles of the biogeochemical-cycle related AMGs in the PRE viruses.

**Additional file 2: Table S1.** Physicochemical data of the PRE samples. **Table S2.** Assembly qualities of the scaffolds/contigs. **Table S3.** Detailed information of the vOTUs identified in the PRE samples. **Table S4.** Detailed information of virus-host linkages prediction. **Table S5.** Annotation and abundance of the lytic viral AMGs. **Table S6.** Annotation and abundance of the temperate viral AMGs. **Table S7.** Occurred frequencies of viruses containing specific AMG functions in the lytic and temperate viral communities. **Table S8.** Normalized expression level of lytic viral AMGs in the free-living (FL) and particle-attached (PA) samples. **Table S9.** Normalized expression level of temperate viral AMGs in the free-living (FL) and particle-attached (PA) samples. **Table S10.** Enriched AMGs (KOs) among three habitats in the lytic or temperate viral communities. **Table S11.** Comparisons of the expression level of functional pathways between free-living (FL) and particle-attached (PA) samples in lytic or temperate viruses. **Table S12.** Number of viruses encoding AMGs that belonged to specific functional pathway in the lytic viruses that could infect the given host order. **Table S13.** Number of viruses encoding AMGs that belonged to specific functional pathway in the temperate viruses that could infect the given host order. **Table S14.** Detailed information about the biogeochemical-cycle related viral AMGs in the PRE samples.

## Data Availability

All sequencing data from the PRE are available at the China National Center for Bioinformation (https://ngdc.cncb.ac.cn/) under the accession number CRA005797 and CRA003857 (metagenome), CRA005800 and CRA003854 (amplicon), and CRA005802 (metatranscriptome). Viral population sequences (larger than 10 kb or circular) in the GOV 2.0 are available through iVirus (https://datacommons.cyverse.org/browse/iplant/home/shared/iVirus).

## Abbreviations

AMG: Auxiliary metabolic gene
KEGG: Kyoto Encyclopedia of Genes and Genomes
KO: KEGG ortholog
PRE: Pearl River Estuary
GOV: Global Ocean Viromes
FL: Free-living
PA: Particle-attached
SE: Sediment
