# Supplementary material for: Viral community-wide auxiliary metabolic genes differ by lifestyles, habitats, and hosts
Source: Microbiome. 2022 Nov 5;10:190. doi: 10.1186/s40168-022-01384-y (PMC9636769; doi:10.1186/s40168-022-01384-y)
Supplement: Supplementary file 2 — Additional file 1: Fig. S1. Geographic distributions of viruses. Fig. S2. Comparisons of the genomic properties between lytic and temperate viruses in the rarefied completeness-filtered dataset. Fig. S3. Comparisons of the functional diversities and compositions of AMGs between lytic and temperate viruses in three datasets. Fig. S4. Lifestyle-dependent AMG compositions in three datasets. Fig. S5. Comparisons of the viral genomic properties and compositions of AMG functions based on GOV 2.0 dataset. Fig. S6. Expression profiles of lytic and temperate viral communities in the rarefied dataset. Fig. S7. Virus-host linkages at phylum level. Fig. S8. Host ranges of the lytic and temperate viruses in the PRE. Fig. S9. Biogeochemical-cycle related AMGs in the PRE viruses. Fig. S10. Expression profiles of the biogeochemical-cycle related AMGs in the PRE viruses. [file 40168_2022_1384_MOESM1_ESM.pdf]

Supplementary figures

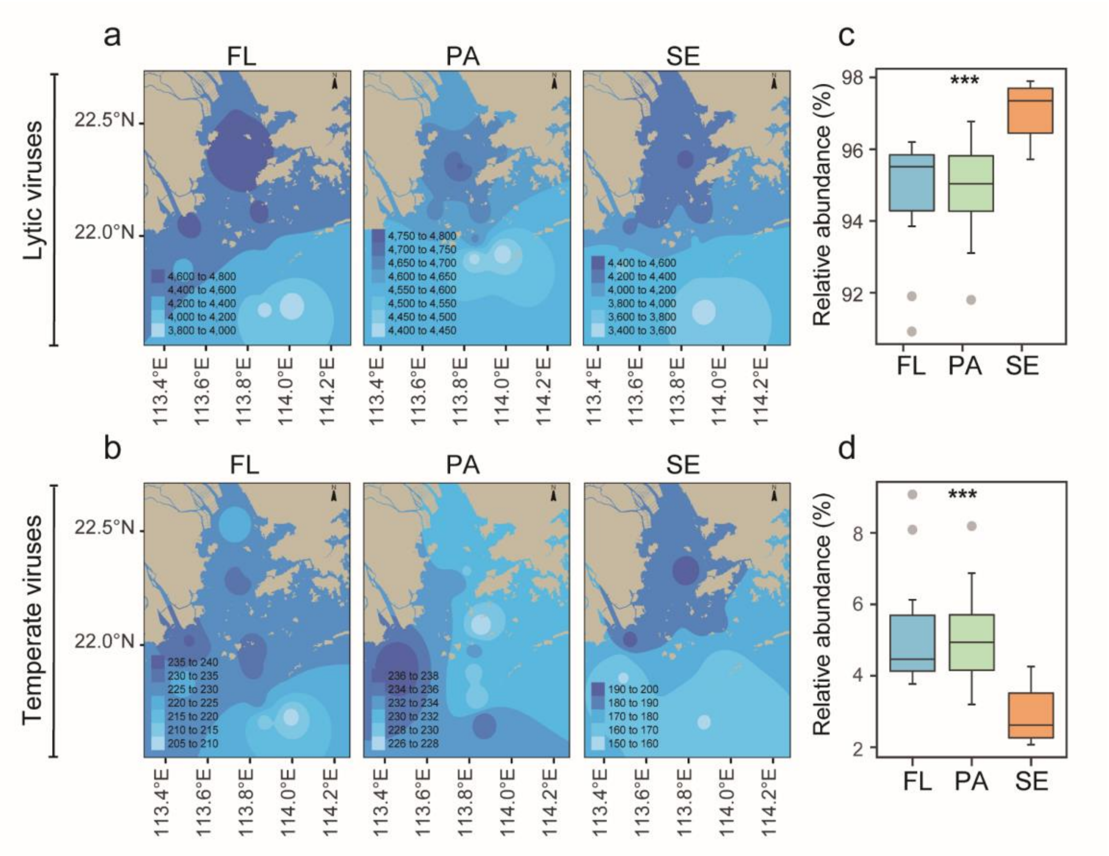

Fig. S1 Geographic distributions of viruses. Diversity distribution of lytic **(a)** and temperate **(b)** viruses in the three habitats (*i.e.*, water, particle, and sediment) of the PRE. Diversity was measured as the vOTU number. Boxplots show the relative abundances of lytic **(c)** and temperate **(d)** viruses in each habitat. The significant difference of the viral relative abundances among three habitats was determined by Analysis of Variance (ANOVA; *** $P < 0.001$) for each lifestyle. FL, free-living; PA, particle-attached; SE, sediment.

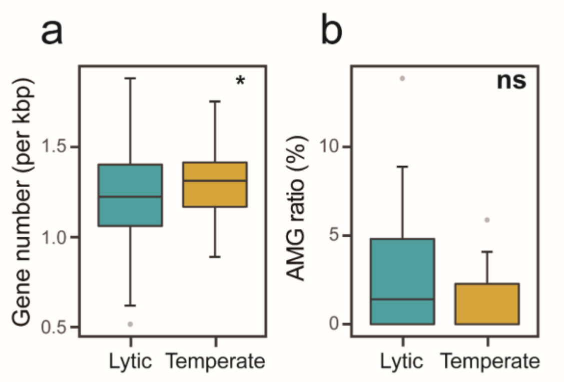

Fig. S2 Comparisons of the genomic properties between lytic and temperate viruses in the rarefied completeness-filtered dataset. Gene densities **(a)** and AMG ratios **(b)** of lytic and temperate viruses. The significance of the differences was determined by Mann-Whitney U test (* $P < 0.05$).

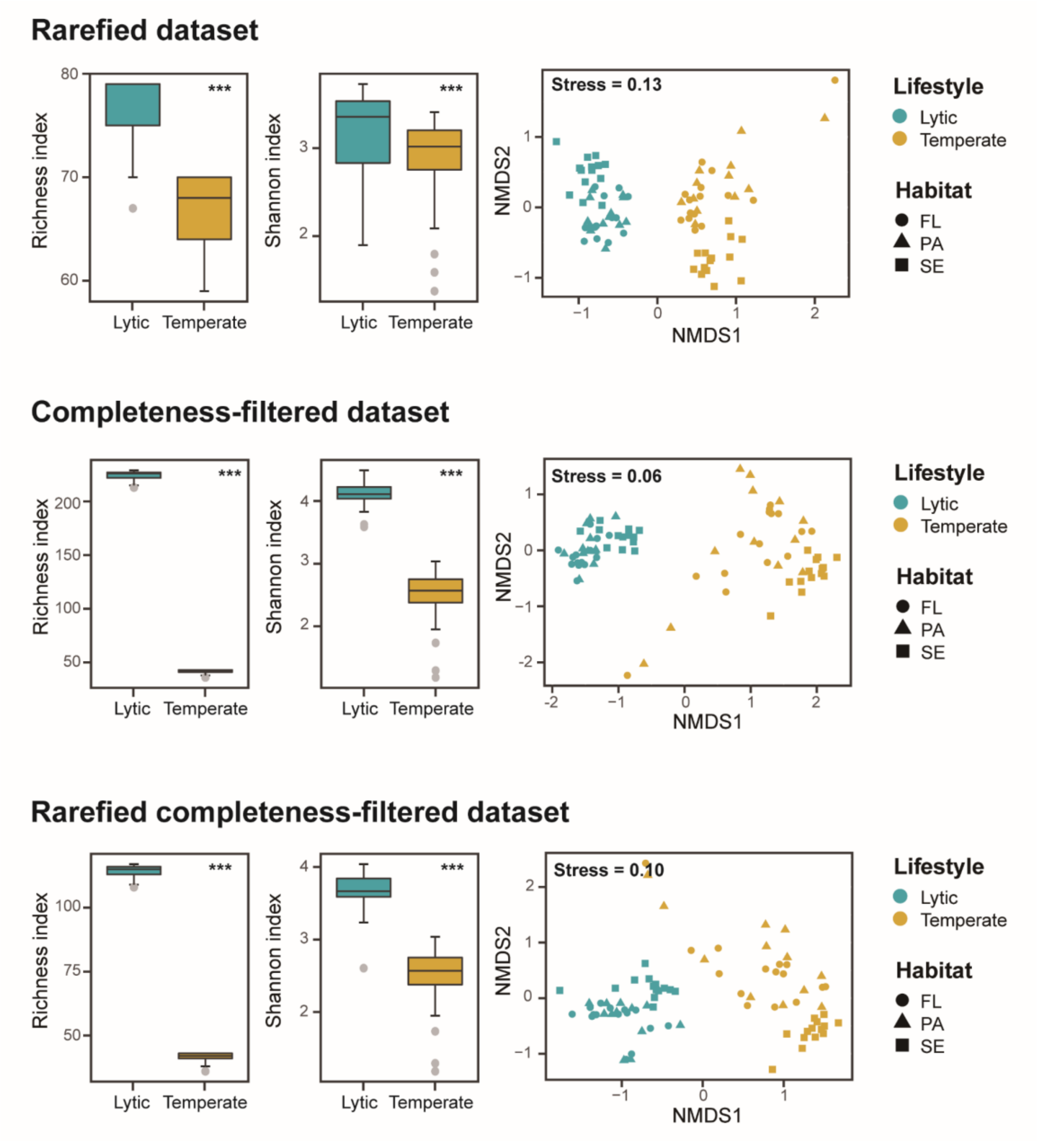

Fig. S3 Comparisons of the functional diversities and compositions of AMGs between lytic and temperate viruses in three datasets. The significance of the differences in functional diversities was determined by Mann-Whitney U test (*** $P < 0.001$). NMDS plots show the dissimilarities of AMG compositions (KOs' relative abundance) between different lifestyles and habitats. FL, free-living; PA, particle-attached; SE, sediment.

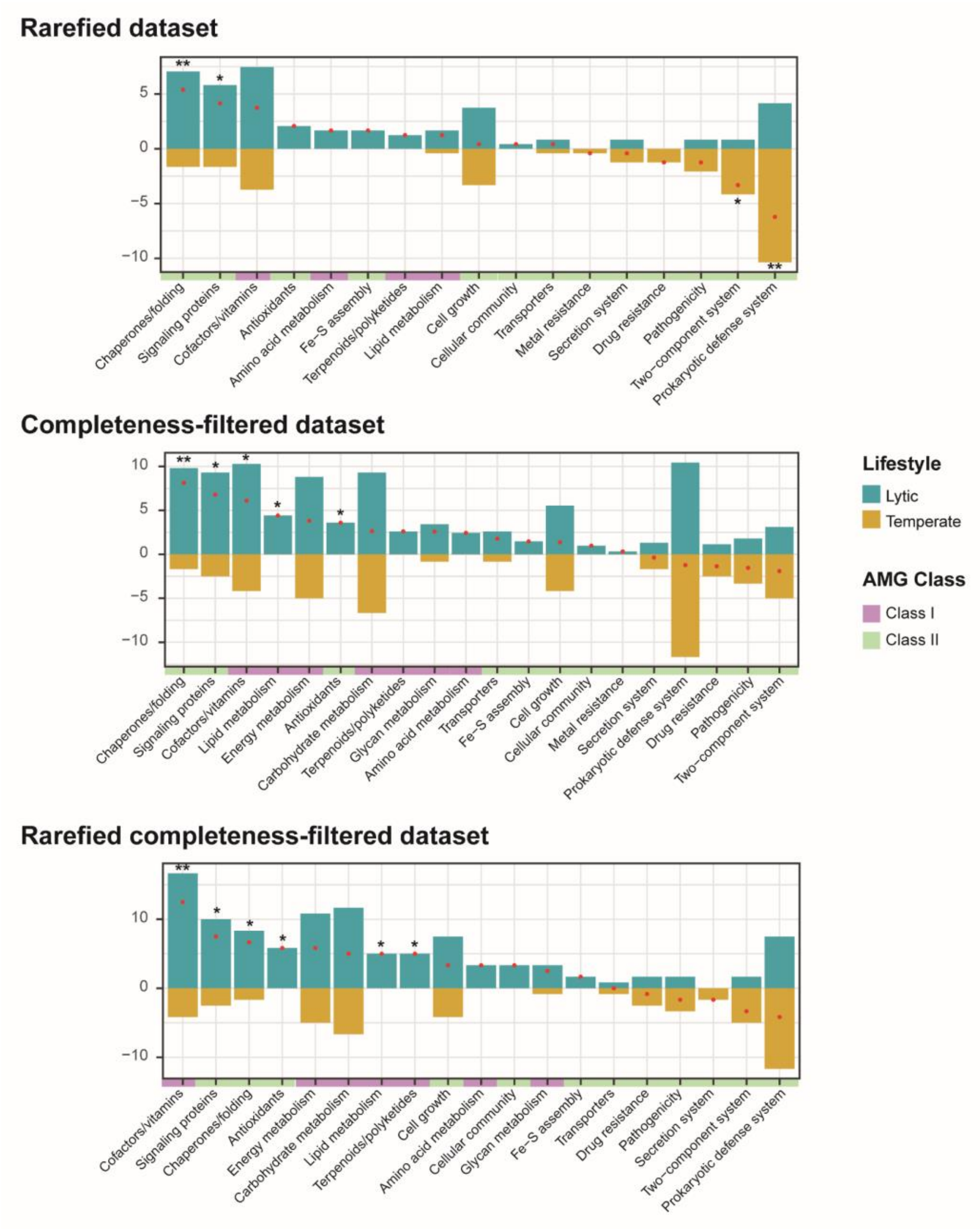

Fig. S4 Lifestyle-dependent AMG compositions in three datasets. The bars show the occurred frequencies of viruses carrying specific function in the whole lytic (blue bars) or temperate (yellow bars) viral communities. Red dots represent the differences of frequency between two lifestyles (lytic minus temperate). The asterisks on top of the bars indicate the statistical significance level (Fisher's test, * $P < 0.05$, ** $P < 0.01$).

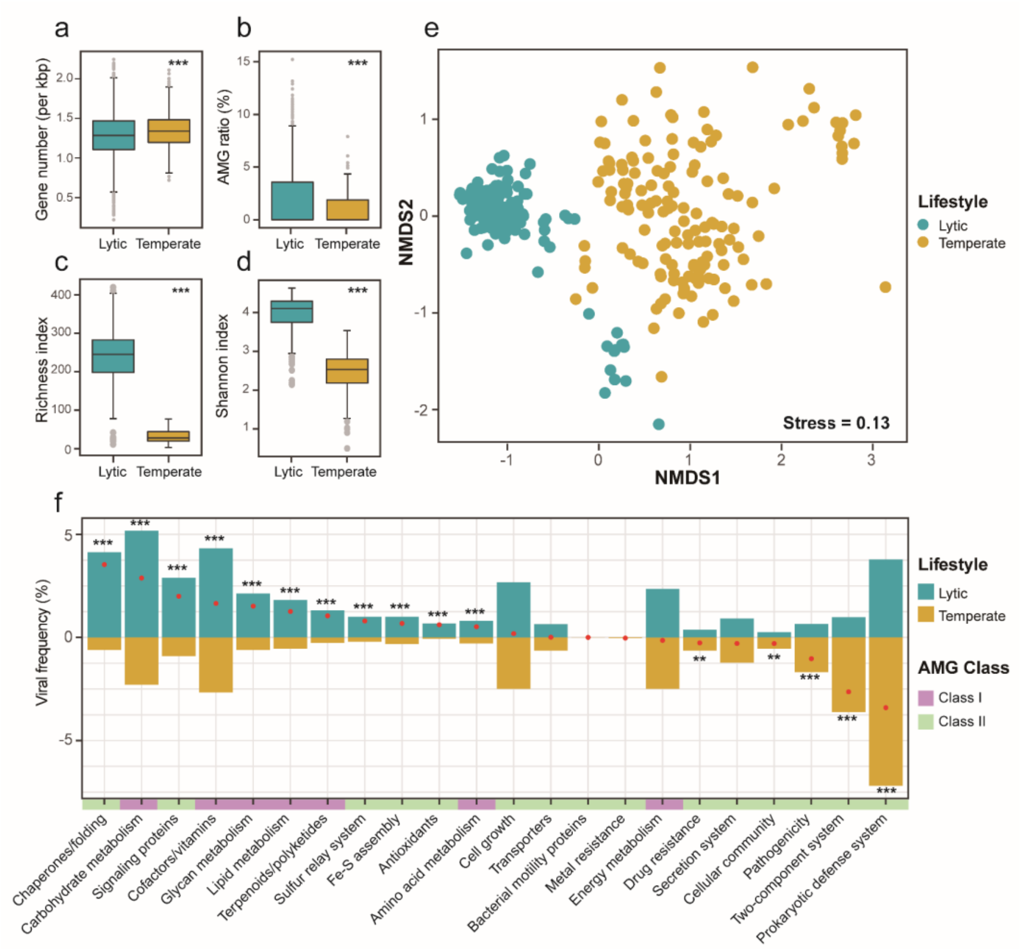

Fig. S5 Comparisons of the viral genomic properties and compositions of AMG functions based on GOV 2.0 dataset. Gene densities **(a)** and AMG ratios **(b)** of high-genome-completeness viral populations, and AMG diversities **(c, d)** of lytic and temperate viral communities. The significance of the differences was determined by Mann-Whitney U test (*** $P < 0.001$). **(e)** NMDS of AMG compositions based on Bray-Curtis dissimilarity of KOs' relative abundance in different samples. **(f)** Occurred frequency of viruses carrying specific function in the whole lytic (blue bars) or temperate (yellow bars) viral communities. Red dots represent the differences of frequency between two lifestyles (lytic minus temperate). The asterisks on top of the bars indicate the statistical significance level (Fisher's test, ** $P < 0.01$, *** $P < 0.001$).

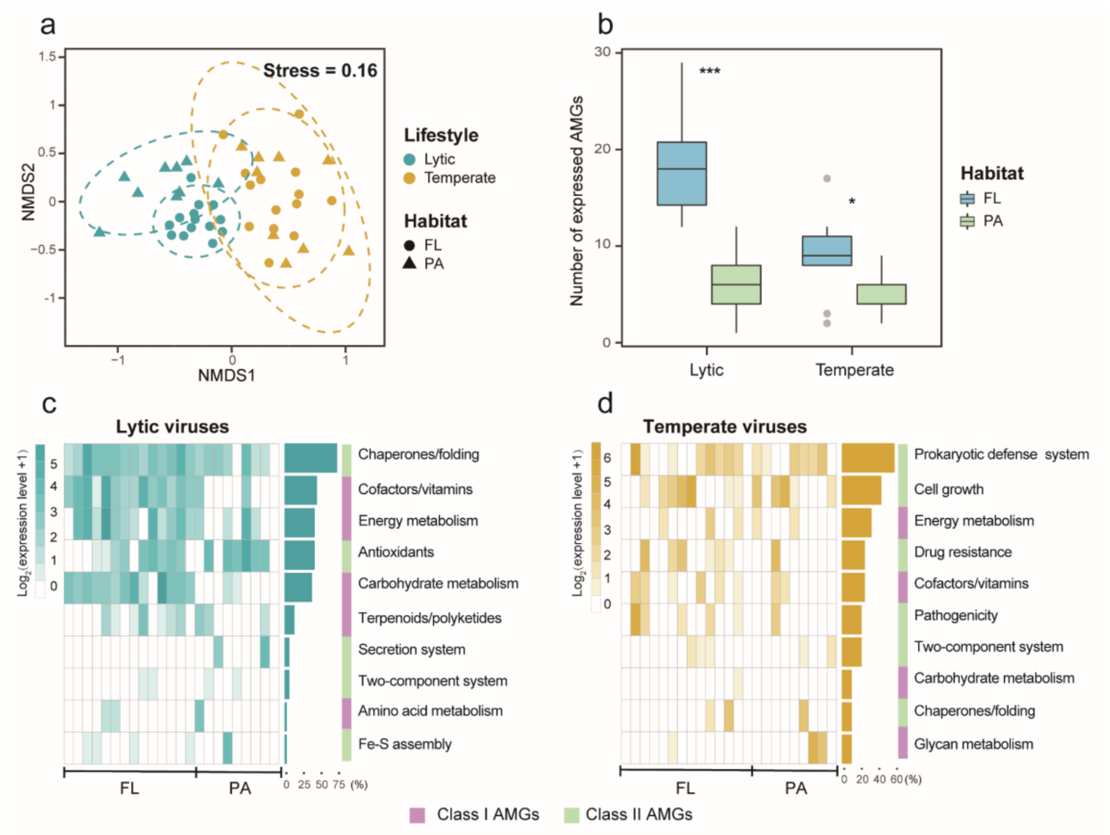

Fig. S6 Expression profiles of lytic and temperate viral communities in the rarefied dataset. (a) NMDS of the expression profiles of viral AMGs at KO level in the FL (free-living) and PA (particle-attached) samples. The expression level of each KO was calculated as the expression abundance (FPKM) divided by gene abundance (FPKM). **(b)** Numbers of active AMGs (KOs) across different lifestyles and habitats. The significant differences between the FL and PA samples were determined by Mann-Whitney U test (* $P < 0.05$, *** $P < 0.001$). Heatmaps of the highly expressed functional pathways in the lytic **(c)** and temperate **(d)** viral communities across different samples. The sidebar lengths represent the relative frequencies of samples, in which the expression level of the given AMG function was in the top five. The right color bars showed the AMGs' functional classes.

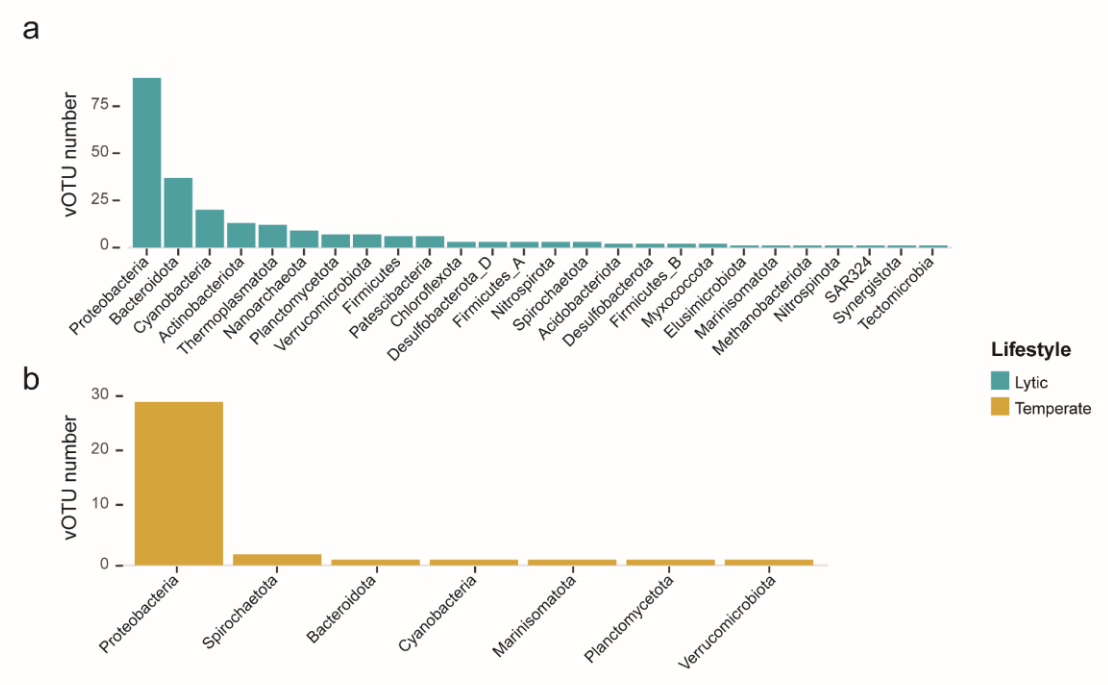

Fig. S7 Virus-host linkages at phylum level. The bars show the vOTU numbers of lytic **(a)** and temperate **(b)** viruses that could infect the given host phylum.

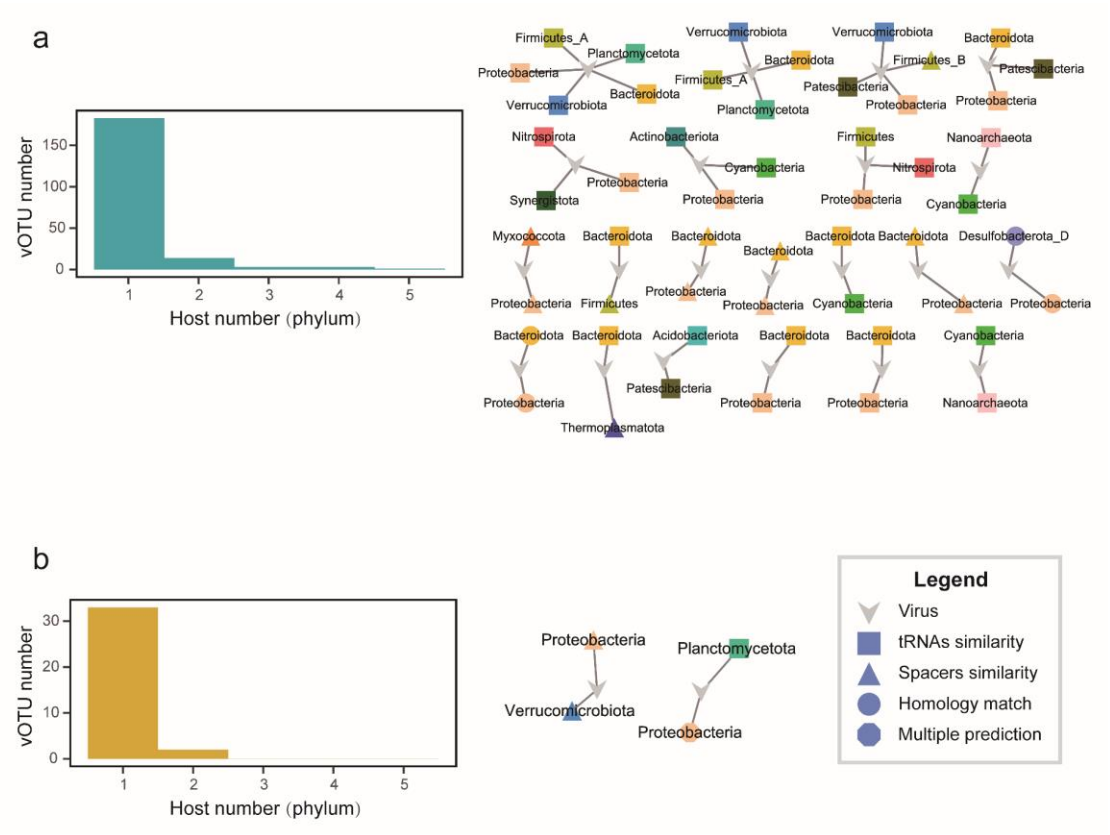

Fig. S8 Host ranges of the lytic and temperate viruses in the PRE. The bars show the frequencies of lytic **(a)** and temperate **(b)** vOTUs that could be linked to a given number of host phylum. Networks show the virus-host linkages of "broad-host-range viruses", and the shape of dots indicates the corresponding host prediction methods.

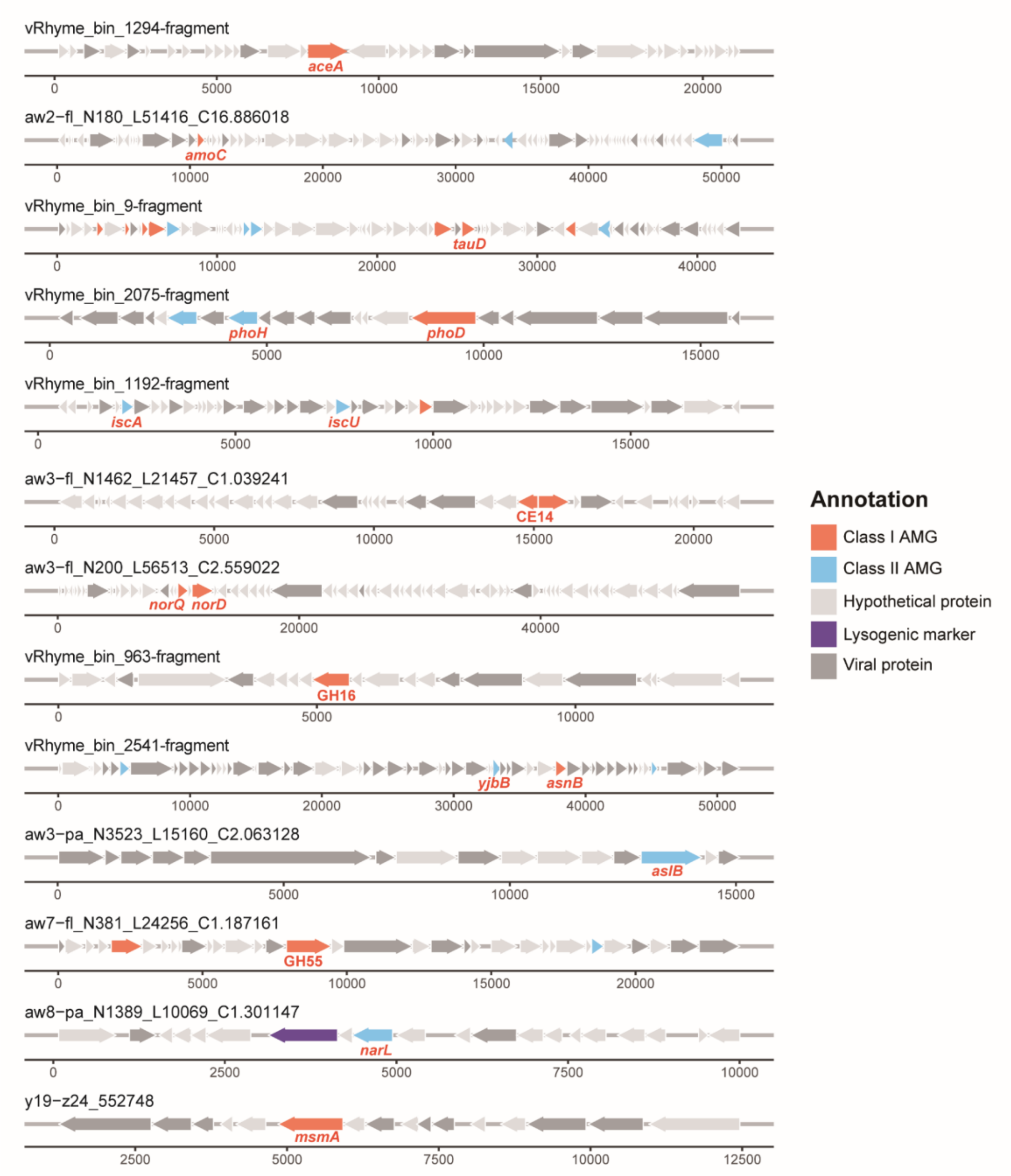

Fig. S9 Biogeochemical-cycle related AMGs in the PRE viruses. Genome maps of some vOTUs that contain AMGs involved in carbon (*aceA*, CE14, GH16, GH55), nitrogen (*amoC*, *norD*, *norQ*, *asnB*, *narL*), phosphorus (*phoH*, *phoD*, *yjbB*), and sulfur (*tauD*, *iscA*, *iscU*, *aslB*, *msmA*) cycles. Genes related to different functions are shown by arrows with different colors in the maps.

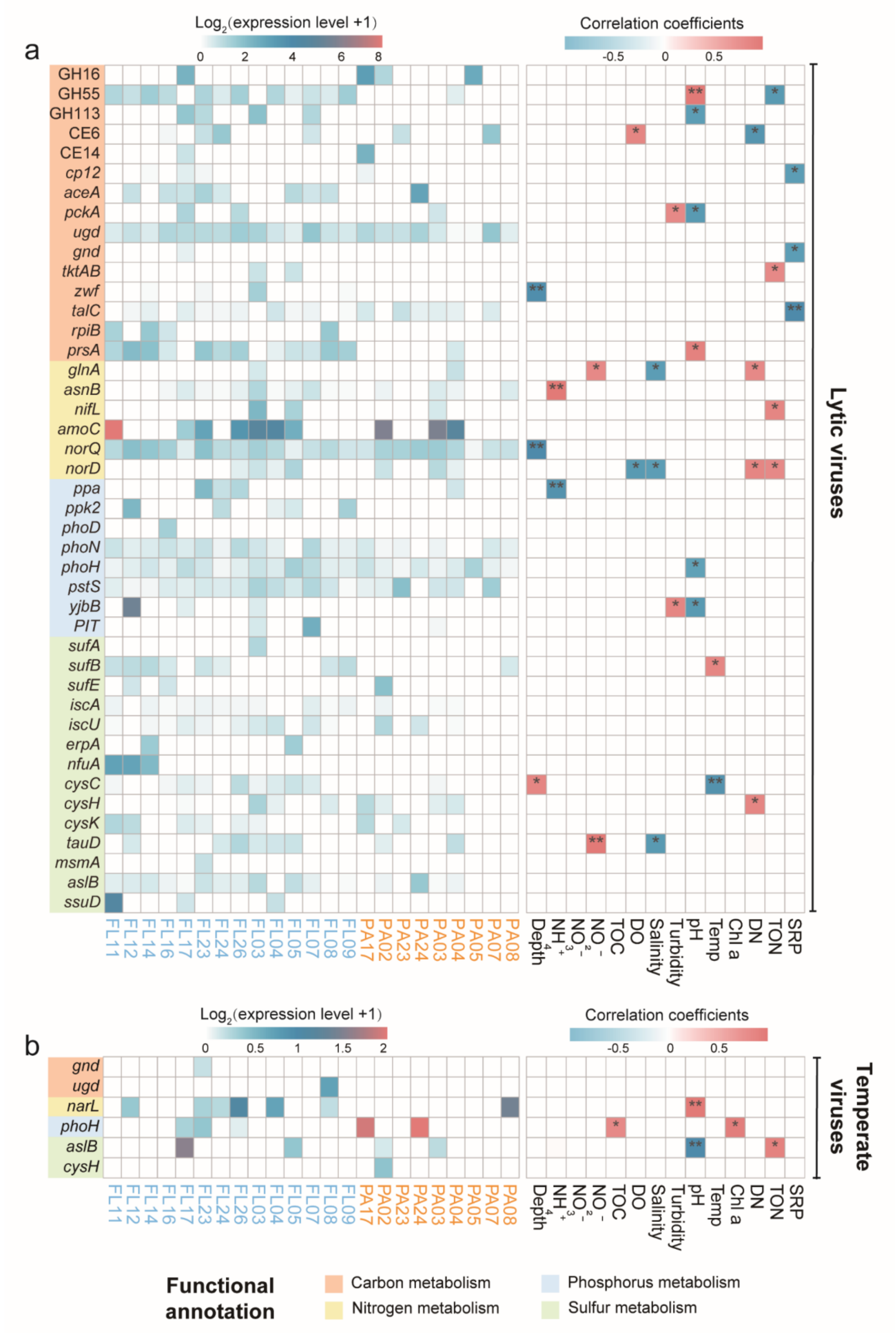

Fig. S10 Expression profiles of the biogeochemical-cycle related AMGs in the PRE viruses. The left heatmaps show the normalized expression level of

biogeochemistry-related AMGs (KOs) of lytic **(a)** and temperate **(b)** viruses in different samples. The expression level of each KO was calculated as the expression abundance (FPKM) divided by gene abundance (FPKM). The right heatmaps show the spearman's correlations between the total expression level (the sum expression level of FL and PA fractions) of specific AMG function (KO) in a given lifestyle and the environmental factors in water samples. The asterisks indicate the statistical significance level (* $P < 0.05$, ** $P < 0.01$). FL, free-living; PA, particle-attached; TOC, total organic carbon; Temp, temperature; Chl a, chlorophyll a; DN, dissolved nitrogen; TON, total organic nitrogen; SRP, soluble reactive phosphate.
